# Monocyte correlates of neurocognitive impairment in chronic HIV infection include early, persistent changes with ART

**DOI:** 10.1172/jci.insight.194766

**Published:** 2026-08-27

**Authors:** Hai Duc Nguyen, Andrew K. Ding-Su, Caroline Soulas, Tricia H. Burdo, Patrick Autissier, Pasiri Sithinamsuwan, Nitiya Chomchey, Jintanat Ananworanich, Victor Valcour, Silvia Ratto-Kim, Woong-Ki Kim, Kenneth C. Williams

**Affiliations:** 1 Division of Microbiology, Tulane National Biomedical Research Center, Tulane University, Covington, Louisiana, USA.; 2 Biology Department, Boston College, Chestnut Hill, Massachusetts, USA.; 3 Innate Pharma, Marseille, France.; 4 Department of Medicine, Rutgers Institute of Translational Medicine and Science, Rutgers Biomedical Health and Sciences, Rutgers University, New Brunswick, New Jersey, USA.; 5 Division of Neurology, Department of Medicine, Phramongkutklao Hospital, Bangkok, Thailand.; 6 SEARCH Foundation, Bangkok, Thailand.; 7 Department of Global Health, Amsterdam Medical Center, University of Amsterdam, Amsterdam, Netherlands.; 8 Department of Neurology, University of California, San Francisco, San Francisco, California, USA.; 9 International Vaccine Institute, Seoul, South Korea.; 10 Department of Microbiology & Immunology, Tulane University School of Medicine, New Orleans, Louisiana, USA.

**Keywords:** AIDS/HIV, Immunology, Neuroscience, Macrophages, Monocytes, Neurological disorders

## Abstract

Persistent monocyte activation contributes to HIV-associated neurocognitive disorders (HAND), yet biomarkers that predict neurocognitive impairment before and after antiretroviral therapy (ART) remain incompletely defined. We evaluated monocyte subsets and activation markers in participants from the SEARCH007 cohort before ART initiation and at 6 and 12 months following treatment. Increased frequencies of CD14^+^CD16^+^ monocytes and elevated CD163 expression were associated with worsening neurocognitive performance and HAND severity. Plasma soluble CD163 levels increased with neurocognitive impairment and correlated with plasma HIV RNA levels, while CCR2 expression was associated with a composite neuropsychological performance Z-score (NPZglobal). Notably, CD169 expression was elevated across all monocyte subsets and demonstrated a stepwise increase with worsening neurocognitive impairment. Although ART reduced overall monocyte activation, elevated CD169 expression persisted in some individuals despite virologic suppression. Bayesian kernel machine regression and random forest analyses identified CD169 expression as one of the strongest predictors of cognitive impairment, surpassing plasma viral load, CD4^+^ T cell count, and several established monocyte activation markers. These findings identify monocyte CD169 expression as a biomarker of neurocognitive dysfunction before and during the first year of ART and support further investigation of its role in HAND pathogenesis.

## Abstract 2

We studies monocyte activation prior to and after ART in PLWH with and without neurocognitive dysfunction and find monocyte markers including CD169 that correlate with dysfunction.

## Introduction

HIV-associated neurocognitive disorders (HAND) affect an estimated 50% of people living with HIV (PLWH) and are associated with legacy factors and non-compliance with antiretroviral therapy (ART), time after HIV infection when ART was initiated, risk-taking behavior, and impaired daily functioning (1–5). Before the ART era, HAND was a subacute disease associated with increased risk of death (6, 7). In the ART era, HAND has become a chronic disease, and its progression no longer correlates with CD4^+^ T cell counts or plasma viral load (8–10). Importantly, early studies demonstrated that at least 50% of individuals had improvement following ART initiation (11–16). Currently, there is no consensus on a biomarker of HAND that can be used in the clinical setting, although expansion of CD14^+^CD16^+^ monocytes, increased expression of CD169 on monocytes, increased levels of soluble CD14 (sCD14) and CD163 (sCD163) in plasma, and increased CCR2 expression on classical monocytes have been considered (17). Diagnosis requires neuropsychological testing, which is expensive and time-consuming and requires specialized training. The identification of a HAND biomarker(s) would enable rapid, scalable stratification of PLWH and provide insight into the mechanisms behind the disease. Moreover, few studies to date have been able to assess monocyte populations and markers before and after ART (less than 1 year) with regard to correlations with HAND. Such studies may provide insight into the mechanisms of HAND.

Although the etiology of HAND is clear, HAND and various HIV-related comorbidities in PLWH are likely to be a result of chronic inflammation and neuronal damage in the central nervous system (CNS) that persist despite ART. In untreated HIV infection, the accumulation of perivascular macrophages in the brain and expansion of the intermediate CD14^+^CD16^+^ monocyte subpopulation correlate with prevalence of HAND. Monocytes in PLWH exhibit distinct transcriptional profiles and phenotypes in comparison with uninfected individuals, including altered surface expression of CCR2, CD40, CD86, CD169, CX3CR1, and HLA-DR, among others (9, 18–24). Although ART normalizes the frequencies of monocyte subsets, sometimes early and transiently, and some HIV-induced phenotypic changes, monocytes continue to exhibit altered expression of surface proteins including CCR2, CD163, CD169, and tissue factor and produce soluble factors including sCD14, sCD163, and MCP-1/CCL2 (19, 21–27).

Phenotypic alterations in activated monocytes and their associated soluble factors are promising candidates for HAND biomarkers in the ART era, especially as they may point to mechanisms of immune activation and chronic inflammation. We have previously shown that levels of plasma sCD163, shed by activated monocytes/macrophages, correlate with the severity of neurocognitive deficit in ART-treated PLWH and predict CNS histopathology in SIV-infected animals with AIDS and SIV encephalitis (28, 29). Levels of sCD14 in plasma, another protein shed by activated monocytes/macrophages, and monocyte-associated markers including HLA-DR, MCP-1, CCR2, and CCR5 have also been associated with impaired cognitive performance in the ART era (8, 30).

Most studies to date have been limited to studying PLWH on durable ART after therapy initiation. These cross-sectional studies may have limited sensitivity for defining biomarkers of HAND and HAND severity as well as markers of ART effectiveness, especially before and early after ART initiation. We tracked ART-naive PLWH before and after 6 and 12 months of ART in the SEARCH007 cohort (hereafter called the Thai cohort), measuring monocyte phenotypes, soluble factors in plasma, and neurocognitive scores (HAND and NPZglobal scores) within and between individuals. These changes were correlated with cognitive function. We asked whether monocyte markers distinguished cognitively impaired individuals from cognitively normal individuals before and after ART. We also asked whether altered monocyte subsets (based on CD14 and CD16), monocyte markers (CCR2, CD44, CD169), or soluble factors of monocytes/macrophages (sCD163) correlated with improvements in cognitive function over time or were predictive of HAND recovery or persistence in individuals starting ART. Additionally, we investigated whether these monocyte markers differentiate between severities of HAND, including asymptomatic neurocognitive impairment (ANI), mild neurocognitive disorder (MND), and HIV-associated dementia (HAD), and NPZglobal scores of cognitive function.

## Results

We studied 30 ART-naive individuals and 7 controls before treatment and at 6 and 12 months after ART initiation. All PLWH participants had CD4 counts of fewer than 350 cells/mm^2^, with detectable plasma RNA ranging from 1,985 to 750,000 copies/mL before ART. The average age was 35 years (range 22–54), with 11 males and 19 females. Participants were grouped by neurocognitive diagnosis: neurologically normal (NL) (*n* = 9), asymptomatic neurocognitive impairment (ANI) (*n* = 6), mild neurocognitive disorder (MND) (*n* = 6), and HIV-associated dementia (HAD) (*n* = 9) (Supplemental Table 1; supplemental material available online with this article; https://doi.org/10.1172/jci.insight.194766DS1). We also studied individuals grouped as asymptomatic (NL and ANI) and symptomatic (MND and HAD). Additionally, we used NPZglobal scores, a composite of individual psychological test scores converted to *z* scores that measure standard deviations of a person’s performance from a normative mean (based on age, education, and demographically matched healthy controls). Controls consisted of HIV-seronegative individuals (*n* = 7; average age 35, range 27–41; 4 male and 3 female). Blood samples and flow cytometric analyses of T lymphocytes and monocytes were conducted before ART and 6 and 12 months after ART initiation.

The neurocognitive status of the participants at the end of the study was as follows: all asymptomatic participants (NL and ANI) remained asymptomatic at follow-up. Six symptomatic patients (MND and HAD) improved to asymptomatic status, while 9 of the 15 impaired participants remained impaired. Thus, the final classification included 14 NL, 7 ANI, 7 MND, and 2 HAD. Correlations of neuropsychological performance (NPZ) composite scores for all HIV^+^ individuals prior to ART indicate an indirect correlation with plasma viral load (Spearman’s correlation *r* = –0.42, *P* = 0.02) (Figure 1A). Analysis at 6 and 12 months after ART demonstrated a significant improvement in NPZ composite (NPZcomp) scores (Figure 1B). The majority of individuals on ART had undetectable plasma virus after 6 and 12 months of ART (Supplemental Table 1). Three ART participants did not have decreased plasma virus (P001, P002, and P020) and were likely non-ART-compliant, while at least 3 individuals had detectable plasma virus at one of the time points after 6 months of ART.

Monocyte subset analysis was performed on frozen and thawed PBMCs using previously described methods and cytometric gates. Of the total cohort, we had complete PBMC samples (pre-ART, 6 months and 12 months post-ART) from 30 individuals that were used for monocyte subset and marker analysis described below. A gating scheme for identifying monocyte subpopulations is shown in Figure 2A. Cell viability, assessed through live-dead cell analysis, exceeded 95% (data not shown). Three monocyte subpopulations were analyzed based on CD14 and CD16 antigens: CD14^+^CD16^–^ (classical), CD14^+^CD16^+^ (intermediate), and CD14^lo^CD16^+^ (non-classical). The flow cytometer was calibrated before all runs, and the data were normalized. We were unable to determine the absolute monocyte cell counts owing to the lack of monocyte counts from individual complete blood count. There was an increased percentage of CD14^+^CD16^+^ monocytes among individuals with HIV, compared with HIV-seronegative individuals. The percentage of CD14CD16 monocytes significantly decreased with 6 months of ART and further decreased after 12 months of therapy, approaching percentages similar to those in uninfected controls (Figure 2C).The percentage of CD14^+^CD16^–^ cells increased after ART, while the percentage of CD14^–^CD16^+^ cells was unchanged (Figure 2, B and D). No monocyte subpopulation correlated with HAND or psychological deficit in ART participants.

Before ART, the percentage of CD14^+^CD16^–^ monocytes correlated directly with plasma viral load (*r* = 0.39, *P* = 0.03) (Figure 2B), whereas the percentage of CD14^+^CD16^+^ monocytes did not correlate. The percentage of CD14^–^CD16^+^ monocytes correlated indirectly with plasma viral load, while the percentage of CD14^+^CD16^+^ cells did not show a correlation (Figure 2C). The percentage of CD14^–^CD16^+^ monocytes indirectly correlated with plasma viral load (*r* = –0.65, *P* = 0.0001) (Figure 2D).

The classical monocytes and non-classical subpopulation are defined as CD14^+^CD16^–^CCR2^+^ and CD14^–^CD16^+^CX3CR1^+^, respectively. CCR2 is thought to promote monocyte extravasation into tissue, whereas CX3CR1 is associated with “patrolling” along blood vessels. CCR2 mean fluorescence intensity (MFI) on CD14^+^ monocytes was lower in HIV-infected individuals compared with uninfected controls. Before ART, CCR2 MFI did not distinguish between HAND categories. CCR2 MFI on classical monocytes increased significantly at 6 months of ART and then decreased from 6 to 12 months after ART initiation (Figure 3A). We observed a direct correlation between the MFI of CCR2 and the NPZglobal score (*r* = 0.39, *P* = 0.033) (Supplemental Figure 1). The MFI of CX3CR1 on CD14^+^CD16^+^ and CD14^–^CD16^+^ monocytes was lower in HIV^+^ individuals compared with HIV^–^ individuals (Figure 3B). Notably, there was a significant decrease in the CX3CR1 MFIs in both the CD14^+^CD16^+^ and CD14^–^CD16^–^ monocyte subsets in participants with HAD. CX3CR1 significantly increased 12 months after ART on CD14^+^CD16^+^ cells (Figure 3B), and CX3CR1 significantly increased on CD14^–^CD16^+^ cells 6 and 12 months after ART (Figure 3C).

Next, we asked whether monocyte-associated markers differentiate PLWH from people living without HIV (PWoH), and HAND categories before and after ART. Further analyses compared neurocognitively asymptomatic (NL and ANI) individuals with neurocognitively symptomatic participants (MND and HAD). CD4, CD86, and MAC387 MFIs were significantly decreased in all 3 monocyte subpopulations at 6 or 12 months of ART (Figure 4, A–C), and CD68 MFI was significantly decreased on CD14^+^CD16^+^ monocytes at 6 and 12 months of ART (Figure 4D). CD163 MFI significantly decreased on both the CD14^+^CD16^+^ and CD14^lo^CD16^+^ monocytes with ART (Figure 4E), while CD44v6 and CCR5 levels did not change on any of the subsets with ART (Figure 4E and data not shown). None of the markers differentiated between HAND groups before ART.

We previously demonstrated that sCD163 levels in plasma correlate with HIV replication in plasma, expansion of CD14^+^CD16^+^ monocytes, and neurocognitive deficits (HAND) in people living with chronic HIV infection on ART (28, 29, 31). In the current study, we found elevated sCD163 plasma levels in ART-naive individuals compared with HIV^–^ controls, consistent with our earlier findings (Figure 5A). Elevated levels of sCD163 were significantly higher in neurocognitively symptomatic compared with asymptomatic participants. Moreover, we observed significantly increased sCD163 levels in neurocognitively symptomatic participants (MND and HAD). After 6 and 12 months of ART, plasma sCD163 significantly decreased (Figure 5B). In ART-naive participants, there was a correlation between plasma sCD163 and plasma viral load, consistent with our prior reports in chronically infected patients (Figure 5C).

Among myeloid markers studied on monocytes and monocyte subsets, the sialoadhesin molecule CD169, like sCD163 described above, appears to be a reliable indicator of HIV activity including viral replication and plasma virus (20, 32). We did not find CD169 expression on monocytes in PWoH, but high levels were present in PLWH (Figure 6 and data not shown). The MFIs of CD169 on CD14^+^CD16^–^ (*r* = 0.60, *P* < 0.001), CD14^+^CD16^+^ (*r* = 0.57, *P* = 0.001), and CD14^lo^CD16^+^ (*r* = 0.66, *P* < 0.0001) monocytes correlated with plasma viral load prior to ART (Figure 6, A–C). The MFI of CD169 on monocyte subsets also negatively correlated with CD4 counts pre-ART (Figure 6, D–F) and NPZglobal scores (Figure 6, G–I).

To further interrogate the relationship between CD169 expression on monocyte subsets and cognitive performance, we analyzed data from 7 individuals (P003, P004, P005, P013, P014, P015, and P016) who achieved viral suppression (plasma viral load < 50 copies/mL) but were diagnosed with ANI, MND, or HAD at 6 and 12 months after ART. These 7 individuals from the cohort were examined because we had frozen PBMCs collected from them at both data points, and they had viral suppression and ANI, MND, or HAD. Although no statistically significant correlation was observed between the CD169 MFI on monocyte subsets and NPZcomp scores in this cohort (Supplemental Figure 2), there were strong negative correlations between CD169 MFI on CD14^+^CD16^–^ (*r* = –0.70, *P* = 0.034), CD14^+^CD16^+^ (*r* = –0.72, *P* = 0.03), and CD14^–^CD16^+^ (*r* = –0.70, *P* = 0.035) monocytes and CD4^+^ T cell count. NPZcomp scores did not correlate with CD4^+^ T cell counts (*r* = 0.12, *P* = 0.75) (Figure 7, A–D). Taken together, in this small group of individuals with suppressed plasma viral load but with cognitive impairment, CD169 levels did not show a direct linear correlation with NPZcomp scores at any of the time points examined after ART.

To further investigate potential nonlinear relationships among CD169 expression on monocyte subsets, CD4^+^ T cell count, plasma viral load, biomarkers previously associated with HAND in PLWH receiving ART (sCD163, CCR2, CD86, CX3CR1, and MAC387), and cognitive performance, we applied a Bayesian kernel machine regression (BKMR) model to data from 30 participants with CD169 measurements obtained at baseline (pre-ART) and at 6 and 12 months after ART initiation. BKMR allows for the identification of key predictors within a complex mixture model by estimating their individual and combined effects on NPZcomp scores. The BKMR model identified CD169 expression on CD14^+^CD16^–^ (posterior inclusion probability [PIP] = 0.721), CD14^+^CD16^+^ (PIP = 0.654), and CD14^lo^CD16^+^ (PIP = 0.660) monocytes among the most important predictors contributing to cognitive impairment within the mixture model (which included CD169 expression, CD4^+^ T cell count, plasma viral load, and monocyte activation markers associated with HAND [sCD163, CCR2, CD86, CX3CR1, MAC387]) in relation to NPZcomp score (Supplemental Table 2). For example, CD169 expression on classical, intermediate, and non-classical monocytes showed higher PIPs than plasma viral load (PIP = 0.339–0.492) and sCD163 (PIP = 0.409–0.620) and comparable or greater PIPs compared with CD4^+^ T cell counts (PIP = 0.463–0.738).

Furthermore, plasma viral load showed no association with cognitive performance, whereas CD4^+^ T cell count showed a weak linear association at baseline (pre-ART). Other markers (CD86, CXCR2, CX3CR1, and MAC387) showed a nonlinear relationship with NPZcomp scores (inverted U-shaped relationship). However, CD169 expression on monocyte subsets was significantly and negatively associated with cognitive performance. When all other factors were held at their median levels, higher CD169 expression on monocyte subsets was associated with a greater decline in NPZcomp scores at baseline (pre-ART) (Figure 7F, Supplemental Figure 3, and Supplemental Figure 4A). Consistent with these findings, heatmap analysis demonstrated distinct CD169 expression patterns between participants with MND/HAD and those with NL/ANI at baseline (pre-ART) (Figure 7G). To further validate our findings, we used random forest, a machine learning technique that combines multiple decision trees, to determine the key features that most strongly influence the model’s predictive accuracy, a concept known as feature importance. In this analysis, CD169 expression on monocyte subsets emerged as the most significant predictor of cognitive impairment, more so than sCD163, CCR2, and other markers shown to associate with HAND at baseline (Figure 7E and Supplemental Figure 5, A–E). We next evaluated biomarker importance at 6 and 12 months after ART among 30 participants. Although CD169 expression was no longer the highest-ranked variable in the BKMR models, it consistently remained among the top predictors based on PIPs and maintained an inverse association with cognitive function (Figure 7F). At 6 months after ART, CD169 expression remained highly ranked (PIP = 0.612–0.790), comparable to plasma viral load (PIP = 0.690–0.788) and CD4^+^ T cell count (PIP = 0.621–0.731), while exceeding sCD163 (PIP = 0.529–0.610). At 12 months after ART, CD169 expression continued to demonstrate importance similar to or greater than that of plasma viral load (PIP = 0.300–0.430), CD4^+^ T cell count (PIP = 0.248–0.413), and sCD163 (PIP = 0.201–0.411), supporting its persistent association with cognitive impairment throughout ART. Similarly, random forest analysis ranked CD169 expression among the top 15 predictors at 6 months and the top 7 predictors at 12 months after ART (Supplemental Figure 5, A–C and data not shown). Notably, at 6 months after ART, plasma viral load and CD4^+^ T cell count ranked sixth and seventh among the top 15 predictors, whereas CD169 ranked 15th. In contrast, at 12 months after ART, CD169 remained a prominent predictor of cognitive performance, ranking seventh overall, whereas CD4^+^ T cell count ranked 13th and plasma viral load was no longer among the top 15 predictors. These results suggest that CD169 expression remains associated with cognitive impairment despite sustained viral suppression.

Analyzing groups by categories of HAND, we found a stepwise increase in CD169 MFI across all 3 monocyte subpopulations, from NL to HAD, before ART. Comparing asymptomatic (NL and ANI) with symptomatic (MND and HAD) groups, significant differences in MFI were observed across all monocyte subsets (Figure 8, A–C). After ART was started, there were significant decreases in CD169 MFI across all 3 subsets, which dropped to levels similar to those in the HIV^–^ cohort within 3 months after ART (Figure 9, A–C). In 2 individuals on ART whose plasma virus did not decrease, likely because of ART non-compliance, CD169 levels, while dipping initially, returned to HIV-infected levels thereafter and were significantly different from those in HIV-infected individuals who were virologically suppressed (Figure 9D and Supplemental Figure 6A). Interestingly, 5 individuals who were neurologically impaired were virologically suppressed (P04, P07, P012, P013, and P016) and had detectable CD169 on their monocytes, suggesting that CD169 may function as a biomarker of cognitive impairment with viral suppression.

The percentage of CD14^+^CD16^–^ monocytes in suppressed and non-virologically-suppressed individuals remained stable at 6 and 12 months after ART initiation (Supplemental Figure 6B). In contrast, the percentage of CD14^+^CD16^+^ monocytes in virologically suppressed and non-suppressed individuals varied at 6 months with both increases and decreases, before declining at 12 months (Supplemental Figure 6C). Meanwhile, the percentage of CD14^–^CD16^+^ monocytes in virologically suppressed individuals increased at 6 months after ART and then stabilized to pre-ART levels by 12 months (Supplemental Figure 6D).

## Discussion

Using a cohort of PLWH initiating ART, we sought to identify changes in monocyte phenotypes or changes that occur longitudinally with HIV infection and correlate with HAND and NPZglobal scores before and after ART. ART-naive participants had significantly decreased monocyte CCR2 and upregulated monocyte CX3CR1 that persisted even after 12 months of ART. Specifically, the classical CD14^+^CD16^–^ monocytes had a significant downregulation of CCR2 and increased expression of CX3CR1. Both CD14^+^CD16^–^ and CD14^–^CD16^+^ monocytes had increased expression of myeloid markers including CD68, CD86, and CD169. Interestingly, CD163 expression was significantly elevated only on CD14^–^CD16^+^ monocytes, strongly correlating with plasma HIV RNA levels. Together, these findings indicate that monocyte dysregulation with HIV infection is not completely restored with durable ART, and such persistent monocyte activation may be a marker of ongoing immune activation that contributes to ongoing immune activation that contributes to neurocognitive impairment.

We found that CCR2 expression on classical monocytes decreased with HIV infection and did not normalize to pre-infected levels with 12 months of ART. These data are consistent with those of Gama et al., who showed decreased monocyte CCR2 expression in PLWH (33). Although decreased CCR2 expression has previously been associated with HAND, CCR2^lo^ cells are thought to produce lower amounts of inflammatory cytokines. It is possible that decreased CCR2 expression on blood monocytes reflects increased rates of proinflammatory CCR2^+^ monocyte extravasation into the brain. CCR2^lo/neg^ cells represent a distinct subpopulation characterized by lower expression of inflammatory cytokines and activation markers compared with their CCR2^hi^ counterparts during HIV/SIV infection. Conversely, CX3CR1 density increased across all monocyte subsets during HIV-1 infection. In chronically HIV-infected, ART-naive (CHI^+^ART^–^) individuals, CX3CR1 density on intermediate and non-classical monocytes was lower in comparison with chronically infected, ART-treated (CHI^+^ART^+^) individuals, while CHI^+^ART^+^ individuals exhibited higher CX3CR1 density than chronically infected, rapid plasma region test–positive, ART-treated (CHI^+^RPR^+^ART^+^) individuals (34). Moreover, the reduction of CCR2-expressing non-classical monocytes has been linked to cognitive impairment in ART-naive PLWH in the Thai cohort (35). In that study, CD86 expression was correlated with the amount of HIV produced by macrophages in vitro (36). In untreated HIV-1 infection, monocyte subsets showed significant changes, including increased frequencies and densities of CD86 and decreased CCR2 expression across all monocyte subsets with lower CCR2 density in inflammatory monocytes (CD14^+^CD16^+^) compared with controls (19). After ART was started, these changes improved, with decreased CD86, increased CCR2 density in inflammatory monocytes, and enhanced CX3CR1 expression in patrolling monocytes (19). We found monocytes had increased expression of activation markers, including CD68, CD86, and CD169. Consistent with our findings, an ex vivo study reported that monocyte-derived macrophages displayed increased expression of CD69 and CD86 and decreased expression of CD68 at 28 days after infection (37). Lastly, CX3CR1 expression distinguished individuals with HAD from HIV-seronegative controls but failed to differentiate between HIV-infected individuals with and without cognitive impairment. These findings suggest that CX3CR1 may be more reflective of HIV infection itself than of cognitive impairment.

Similar to our findings in PLWH, Tuluc et al. found that decreased CD163 expression on macrophages results in a significant reduction in HIV infection (38). This study, similar to our findings, observed that CD163 expression was significantly elevated on CD14^–^CD16^+^ monocytes and strongly correlated with plasma HIV RNA levels. We also observed higher sCD163 levels in plasma in ART-naive PLWH compared with PWoH, particularly in those with cognitive deficits. sCD163 levels significantly decreased after 6 and 12 months of ART, correlating with plasma viral load pre-ART, confirming earlier findings. A cross-sectional study conducted in Thailand and Cambodia indicated that increased percentages of CD14^+^CD16^+^CD163^+^ and CD14^lo^CD16^+^CD163^+^ monocytes were associated with better neuropsychological test scores (verbal intelligence quotient and performance intelligence quotient [CT2]) in children living with HIV (39). Furthermore, a cross-sectional study reported that plasma levels of sCD163 were positively correlated with the percentage of CD14^+^CD16^+^ monocytes and activated CD8^+^HLA-DR^+^CD38^+^ T cells, while showing an inverse correlation with CD163 expression on CD14^+^CD16^+^ monocytes, and ART effectively reduced sCD163 levels alongside declining HIV RNA (31). In contrast, an ex vivo study found that CD163 expression on CD14^+^CD16^+^ intermediate monocytes from PLWH was notably higher in comparison with PWoH controls. There was also a trend toward increased expression on CD14^+^CD16^–^ monocytes, potentially influenced by HIV-1 therapy, especially therapy involving protease inhibitors (31). Overall, these findings suggest that CD163 expression levels on monocytes could serve as a potential biomarker indicating the immune system’s attempts to mitigate immune activation and inflammation in PLWH, particularly those with HAND.

We also found that CD169 expression on monocytes correlated with the response to ART in terms of plasma virus and correlated positively with the degree of HAND and NPZ scores. CD169, also known as Siglec-1, is a sialic acid–binding immunoglobulin-like lectin predominantly expressed on activated monocytes and macrophages. Its expression is significantly upregulated by type I interferons (IFNs), particularly during viral infections and in lymphoid tissues under conditions of heightened immune activation (40–42). Thus, CD169 likely plays a crucial role in the immune response enhancing the capture of sialylated pathogens, including HIV, facilitating presentation of viral antigens to T cells (32, 43, 44). Through these mechanisms, CD169 can likely amplify anti-HIV viral responses (including innate IFN responses), potentially making it a key correlate in the immunopathogenesis of HIV-associated comorbidities including HAND. Although CD169 has been recognized as a marker of HIV-1 infection and linked to immune activation (20), few studies have examined its association with monocytes and HAND. Previous studies, including those involving the SEARCH007 cohort, have primarily focused on the association of CD169 with general markers of immune activation, rather than its specific involvement in neurocognitive outcomes (35, 45–47).

Our study provides evidence that CD169 expression is not only elevated during HIV infection but is also associated with HAND. Specifically, we observed a stepwise increase in CD169 expression that correlated with worsening neurocognitive performance, suggesting that CD169 may reflect ongoing neuroinflammation and immune dysregulation within the CNS. This association underscores the complexity of immune activation in HIV infection and its potential contribution to neurocognitive impairment. Although ART is known to reduce systemic immune activation, elevated CD169 expression persisted in some individuals with HAND at 6 and 12 months after ART initiation. This persistence suggests that CD169 may be associated with residual immune activation that is not fully resolved by ART. Consistent with our previous findings for sCD163, the timing of ART initiation appeared to influence monocyte CD169 expression, with differences observed between individuals who initiated ART within 1 year of HIV infection and those who initiated treatment later (29). Together, these findings support the potential utility of CD169 as a biomarker of ongoing neuroinflammatory processes associated with HAND and suggest that it may help identify individuals at risk for persistent neurocognitive dysfunction despite virologic suppression. However, these findings should be interpreted with caution, as this study included a relatively small cohort of 30 PLWH and control participants, warranting validation in larger, independent cohorts.

Our study adds to the existing literature examining CD169 expression across monocyte subsets in both ART-naive and ART-treated PLWH. Prior studies have predominantly focused on ART-naive cohorts, limiting the understanding of how treatment modulates monocyte activation over time (35, 45–47). By including both pre- and post-ART cohorts, we were able to evaluate longitudinal changes in CD169 expression and its association with virologic control and neurocognitive outcomes. Interestingly, while CD169 levels decreased in individuals with effective virologic suppression, they remained elevated in PLWH with cognitive impairment, reinforcing the idea that neuroinflammation persists despite ART-mediated viral suppression. In contrast to earlier studies that categorized PLWH into broad cognitive groups (NL, ANI, MND, and HAD), we also distinguished between symptomatic and asymptomatic individuals (35, 45–47). Additionally, we incorporated NPZ scores for a quantitative measure of cognitive function, enabling us to draw stronger correlations between CD169 expression on monocyte subsets and the degree of neurocognitive impairment. This quantitative approach may enhance our understanding of the relationship between CD169 and cognitive function in PLWH but also provides a more reliable metric for future studies investigating HAND biomarkers.

Importantly, our findings extend beyond simple correlations between CD169 expression and cognitive performance. By applying complementary machine learning approaches, including Bayesian kernel machine regression and random forest analyses, we evaluated CD169 within a broader biomarker framework that included plasma viral load, CD4^+^ T cell count, and established monocyte activation markers associated with HAND, including sCD163, CCR2, CD86, CX3CR1, and MAC387. These methods account for complex, nonlinear interactions among correlated biomarkers while identifying variables that contribute most strongly to cognitive outcomes. Across analyses, CD169 expression on monocyte subsets consistently emerged as one of the strongest predictors of cognitive impairment and remained highly ranked throughout ART, even when compared with biomarkers previously linked to HAND. Notably, CD169 retained its predictive importance at 6 and 12 months after ART initiation despite effective viral suppression, suggesting that monocyte activation reflected by CD169 expression may capture persistent pathogenic processes not fully explained by plasma viral load or CD4^+^ T cell count alone. Collectively, these findings support CD169 as a robust biomarker of neurocognitive dysfunction across multiple stages of HIV infection and treatment prior to and within a year of ART.

In addition to the notion of CD169 functioning as a biomarker of HAND as suggested by our work here, and proposed previously by Pulliam, who proposed that CD169 could serve as a marker distinguishing between PLWH with high and low viral loads, particularly among those undergoing ART (48), the mechanistic role of CD169 in HIV pathogenesis and neuroinflammation cannot be overlooked. Recent evidence suggests that CD169 may play a functional role in the IFN antiviral response by facilitating viral entry into monocytes and macrophages, where HIV can establish reservoirs (42). This aligns with our findings of persistent CD169 expression in patients with HAND. Given that IFN-driven pathways are crucial in chronic HIV infection, future research should aim to elucidate the specific signaling mechanisms through which CD169 modulates immune responses in CNS, and how these processes contribute to the development and progression of HAND. Taken together, our study identifies CD169 as a promising biomarker for HAND, providing novel insights into the immune mechanisms underlying HIV-related neurocognitive impairment. By assessing CD169 expression across different monocyte subsets and comparing its levels in ART-naive and ART-treated PLWH, we offer a comprehensive view of its role in the context of HIV infection and neurocognitive outcomes.

In summary, our study highlights the persistent dysregulation of monocyte subsets in people living with HIV, even following the initiation of ART. The identification of CD169 as a biomarker associated with HAND underscores its potential relevance to the immune mechanisms underlying neurocognitive impairment in this population. Our findings demonstrate that CD169 expression is associated with cognitive performance and remains an important predictor of neurocognitive dysfunction during the first year of ART, suggesting that it may reflect ongoing immune activation despite virologic suppression. These results support the utility of CD169, together with other monocyte activation markers, for improving our understanding of the pathophysiology of HAND and identifying individuals at risk for persistent neurocognitive impairment. We note that our data do not support CD169 expression on monocytes as a stand-alone marker, but instead point to activated monocytes/macrophages even with ART in neurocognitive function with HIV. Further studies in larger cohorts are warranted to validate these findings and determine whether CD169 may serve as a useful biomarker for monitoring disease progression, therapeutic responses, and residual neuroinflammation in people living with HIV.

## Methods

*Sex as a biological variable*. While our study included both male and female PLWH, sex was not analyzed as a biological variable.

*Cohort*. This study recruited 50 ART-naive PLWH from the SEARCH007 study (NCT00777426, ClinicalTrials.gov) at the Thai Red Cross AIDS Research Center in Bangkok, Thailand. Participants were eligible for enrollment based on Thai Ministry of Health guidelines for initiating therapy at the time of enrollment (2008 to 2013), which included symptomatic HIV infection or a CD4^+^ T cell count below 350 cells/mm^3^ (49). Cognitively normal (NL) individuals were matched to HAND participants based on factors such as CD4^+^ T cell count, educational level (less than high school, high school, some college, or college degree), sex, and age (within 10 years). Additionally, 10 HIV-uninfected controls were included for comparison. PLWH were classified as either NL or diagnosed with HAND. Among the HAND group, 16 participants met criteria for ANI, 12 for MND, and 9 for HAD, using established clinical assessments and neuropsychological testing protocols (14, 50). The clinical evaluation involved a comprehensive neuropsychological test battery (51), the International HIV Dementia Scale (52), a neurological examination, and brain structural imaging. Final cognitive diagnoses were confirmed through a consensus process involving an HIV neurologist and a neuropsychologist, using standard diagnostic criteria (14). PWoH were selected using the same inclusion and exclusion criteria, with the exception of CD4 count. Exclusion criteria for all participants included prior exposure to ART, positive hepatitis C serology, and conditions known to affect cognitive function (e.g., history of head injury, learning disabilities, major depression, illicit drug use, active opportunistic infections, or past or current CNS infections). A negative urine drug test was also required prior to enrollment. If necessary, lumbar punctures and brain MRIs were performed to rule out CNS opportunistic infections. Informed consent was obtained from all participants before the study. Volunteers returned to the clinic every 3 months over a 1-year period. Peripheral blood mononuclear cells (PBMCs) were collected, and neuropsychological assessments were conducted at baseline, with additional evaluations at 6, 9, and 12 months after enrollment and after ART initiation. For the neurocognitive study, we focused on data collected at baseline and 12 months after the start of ART, analyzing participants according to their initial HAND diagnosis (50).

*NPZglobal score*. Participants underwent a 1-hour neuropsychological testing battery. Raw scores were converted into standardized *z* scores based on Thai normative data, as previously described. The global performance score (NPZglobal) was calculated by averaging of the *z* scores from all tests, with domain-specific scores similarly derived by averaging of the relevant tests for each domain. The neuropsychological battery assessed various cognitive functions, including attention, concentration, working memory, executive function, verbal and visual learning and memory, psychomotor skills, manual dexterity, motor abilities, language, and visuospatial domains, as detailed by Schifitto et al. (50).

*Response to ART*. Participants were categorized as responders and non-responders based on their clinical outcomes following the initiation of ART. Responders were defined as individuals who exhibited significant clinical improvement, characterized by a marked decrease in plasma viral load and/or enhancement in neurocognitive performance, as assessed by NPZ composite (NPZcomp) scores. Conversely, non-responders were identified as individuals who did not show significant clinical improvement after ART, evidenced by unchanged or worsening plasma viral load and/or neurocognitive performance. This classification facilitated the analysis of the effects of ART on the percentage of monocyte subsets based on CD14 and CD16, and expression of myeloid markers (CCR2, CX3CR1, CD44v6, CD86, CD163, and CD169) and their correlation with neurocognitive outcomes.

*Phenotypic characterization of blood leukocytes by flow cytometry*. PBMCs were isolated from the leukapheresis or blood draw product using Ficoll-Hypaque density gradient centrifugation and were subsequently cryopreserved in liquid nitrogen (using a freezing medium composed of RPMI 1640, 10% fetal calf serum, and DMSO) until needed. Immunophenotyping to assess monocyte subset and monocyte-associated surface markers by flow cytometry was conducted on rapidly thawed PBMCs. PBMCs and monocyte subsets were evaluated using a panel of antibodies, including anti-CX3CR1 (clone 2A9-1), anti-CCR2 (clone 48607), anti-CD169 (clone 7-239), anti–HLA-DR (clone Immu-357), anti-CD86 (clone IT2.2), anti-CD163 (clone GHI/61), anti-CD16 (clone 3G8), anti-CD44v6 (clone VFF-7), anti-CD3 (clone SP34-2), anti-CD20 (clone L27), anti-CD14 (clone M5E2), anti-CD4 (clone S3.5), and Live/Dead (catalog L34957). Antibodies were sourced from BD Pharmingen and Invitrogen. The stained cells were washed with phosphate-buffered saline containing 2% fetal bovine serum and then resuspended in freshly prepared 1% paraformaldehyde before analysis using a BD FACSAria II flow cytometer (BD Biosciences), as previously described (53). A total of 100,000 to 300,000 events were collected for analysis. The assessment of CD14 expression used both isotype and population gating strategies, where the lymphocyte population and the isotype controls defined the lower limit for distinguishing CD14^+^ from CD14^–^ cells. The upper limit for CD14^+^ and lower limit for CD14^++^ expression was determined by the CD14^++^ population. CD16 expression was evaluated using a conservative isotype gating approach, corroborated by the fluorescence intensity observed in the CD16^–^ lymphocyte population. Fluorescence minus one (FMO) and isotype gating strategies were used to identify the expression of activation markers. The data were analyzed using FlowJo software (version 10.10, Tree Star).

*Plasma sCD163*. Plasma concentrations of sCD163 were assessed in duplicate using an enzyme-linked immunosorbent assay (ELISA) previously described in the literature (54). Briefly, each assay included control samples and standards that were traceable to purified CD163. The assay demonstrated a total imprecision of less than 6%, with a detection threshold of below 0.006 mg/L. The established reference range for healthy individuals is between 0.89 and 3.95 mg/L (55). All assays were run on coded samples with no individual identifiers.

*Plasma viral RNA*. Plasma HIV-1 RNA levels were measured using the COBAS AmpliPrep/COBAS TaqMan HIV-1 test (Roche Diagnostics), following the manufacturer’s guidelines. RNA levels in the plasma were classified as undetectable if they were below 50 copies/mL.

*Statistics*. The data were analyzed using GraphPad Prism version 10.3.1 (GraphPad Software Inc.) and RStudio version 3.5.3 (RStudio Inc.). Participant demographic and clinical data are expressed as categorical variables and as median with interquartile range for continuous variables. Flow cytometry data were examined with FlowJo version 10 and subsequently exported for additional analysis in GraphPad Prism version 10.3.1. Normality was assessed using the Shapiro-Wilk test. Two-tailed Student’s *t* tests were used for comparisons between 2 groups. One-way or 2-way ANOVA, as appropriate, followed by Tukey’s multiple-comparison test, was used for comparisons involving 3 or more groups. Non-parametric Spearman’s correlation analysis was used to assess associations between biomarkers and clinical variables. A 2-sided *P* value of less than 0.05 was considered statistically significant.

To investigate the nonlinear relationships between cognitive decline (NPZcomp score) and CD169 expression on monocyte subsets, and to compare the impact of CD169, plasma viral load, and CD4^+^ T cell count, we applied BKMR. BKMR is a supervised, non-parametric method that uses kernel functions to model the relationship between multiple predictors and outcome variables. This approach accommodates potential nonlinear associations while also allowing for the inclusion of additional covariates in a parametric manner (56). BKMR was chosen for its ability to capture complex interactions and non-linearities among predictors, as well as its capacity to incorporate prior knowledge and uncertainty into the analysis. Given the potential for nonlinear interactions and multicollinearity among variables, BKMR is particularly advantageous in this context (56). Unlike linear regression, which can fail to detect nonlinear relationships among cognitive decline and related factors (such as CD169 expression on monocytes, plasma viral load, and CD4^+^ T cell count), BKMR can simultaneously account for these complexities without requiring pre-specification of interaction terms (56–58). BKMR’s flexibility makes it ideal for studies like ours, in which multiple interacting factors (e.g., CD169 expression, plasma viral load, CD4^+^ T cell count, and other markers) likely contribute to cognitive decline.

### Our BKMR model was generated using the following equation:

Yi = *h*(CD169 expression on monocyte subsets_i, plasma viral load_i, CD4^+^ T cell count_i, and other markers_i) + β*Z*_i (age, sex) + ϵ_i

Here, the function *h* represents the nonlinear relationships among the predictors (CD169 expression, plasma viral load, and CD4^+^ T cell count, and other markers including CD86, CCR2, CX3CR1, sCD163, and MAC387). *Z* = *Z*_1_, *Z*_2_, …, *Z_q_* represents *q* potential confounders, including age and sex. Before analysis, missing values in exposure variables were imputed using the median value of each respective variable, while missing covariate data were handled by excluding incomplete observations. Continuous biomarker variables were then log_2_-transformed (or standardized, if applicable) to reduce skewness and improve comparability across variables. A Gaussian kernel function was used, and a component-wise variable selection strategy was used. After fitting the model using Markov chain Monte Carlo sampling with a burn-in of 20,000 iterations, we obtained posterior inclusion probabilities (PIPs) for each predictor to identify the most influential factors related to cognitive decline. A PIP threshold of 0.5 was used to determine the importance of each factor (59). Univariate exposure-response functions (95% CI) were calculated to visualize the relationship between selected factors and cognitive decline, while fixing the values of other factors at their median levels. To further validate our findings, we applied random forest modeling using the RandomForests package (version 4.6-14) in R (60). The ntree parameter was set to 500 to identify significant factors, including CD169 expression on monocyte subsets, plasma viral load, CD4^+^ T cell count, and other markers (sCD163, CCR2, CD86, CX3CR1, MAC387), in relation to cognitive impairment.

#### Study approval.

All participants provided written informed consent. This study was approved by the Research Ethics Review Committee for Research Involving Human Participants, Chulalongkorn University, Bangkok, Thailand, the Human Subjects Protection Branch, Walter Reed Army Institute of Research, Silver Spring, Maryland, USA, and the Human Research Protection Office at UCSF.

#### Data availability.

All data that was used for research, including data points for Figures 1–9, can be found in this publication and the supplemental Supporting Data Values file. Data can also be obtained upon request.

## Author contributions

All authors made substantial contributions to the intellectual content of the paper and approved the final version of the manuscript. VV, SRK, CS, THB, JA, and KCW were involved in study design. PS and NC performed neurologic and psychological assessment. HDN, CS, PA, AKDS, and THB were involved in data acquisition and analysis. HDN, AKD, CS, THB, JA, VV, WKK, and KCW were involved in writing the manuscript. Funding was acquired by KCW and SRK.

## Conflict of interest

The authors have declared that no conflict of interest exists.

## Funding support

This work is the result of NIH funding, in whole or in part, and is subject to the NIH Public Access Policy. Through acceptance of this federal funding, the NIH has been given a right to make the work publicly available in PubMed Central.

- NIH grants R01NS040237 (to KCW) and R01NS126091 (to KCW and WKK).
- NIH grant R21MH072388 (to SRK).

## Supplementary Material

Supplemental data

undefined

## Figures and Tables

**Figure 1:**
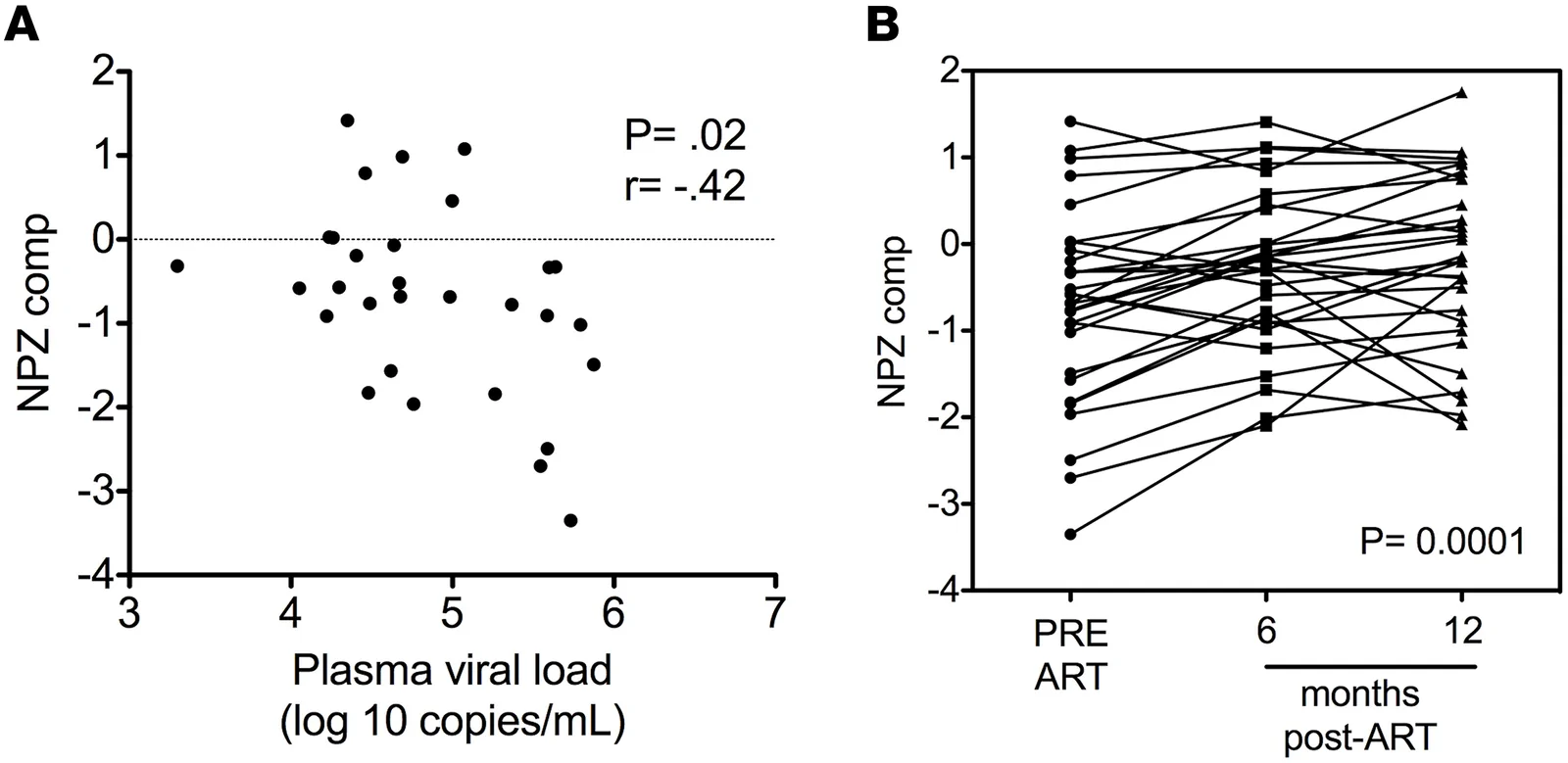
NPZ composite score correlation and response to ART. (**A**) Spearman’s correlation between NPZ composite scores and plasma viral load in 30 ART naive individuals before and 6 and 12 months after ART (*r* = –0.42, *P* = 0.02). (**B**) Changes in NPZ composite scores from baseline (pre-ART) to 6 and 12 months after ART, indicating significant improvement in neurocognitive performance over time. Statistical significance between time points was assessed using 1-way ANOVA with Tukey’s post hoc test for multigroup comparisons. *P* values are indicated for comparisons between each time point. The significant increase in NPZ scores suggests enhanced neurocognitive function with effective ART.

**Figure 2:**
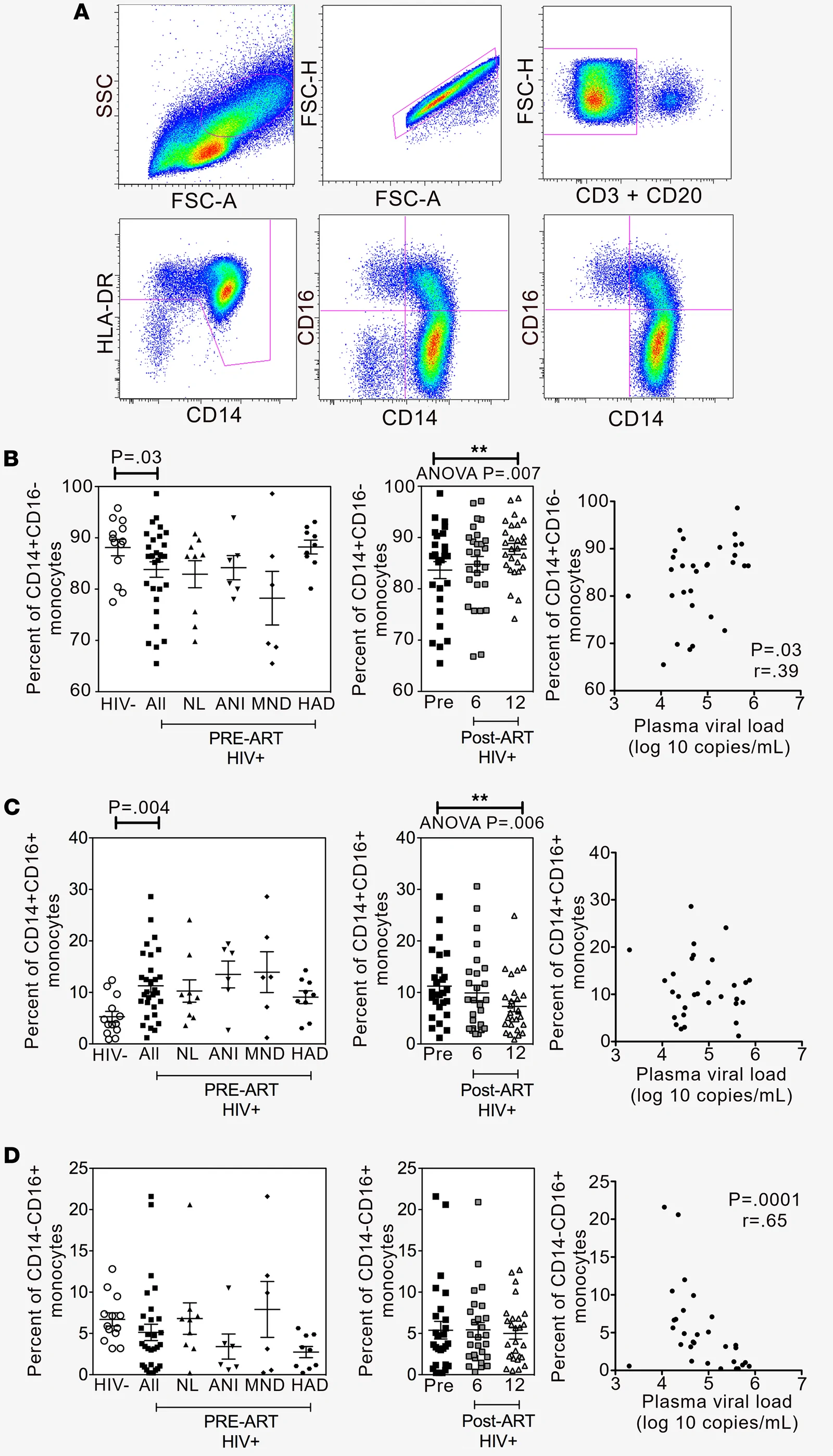
Monocyte subset analysis before and after ART. (**A**) Gating strategy used to identify monocyte subsets: CD14^+^CD16^–^ (classical), CD14^+^CD16^+^ (intermediate), and CD14^–^CD16^+^ (inflammatory) monocytes. Top (left to right): selection gate for cells followed by live cell capture and lineage selection excluding CD3 and CD20 lymphocytes. Bottom (left to right): positive selection of monocytes by HLA-DR and CD14, and analysis of monocytes based on CD14 and CD16 staining. (**B**–**D**) Percentage of classical, intermediate, and inflammatory monocytes before and after ART in PLWH and PWoH. (**B** and **C**) Significant increase in CD14^+^CD16^–^ monocytes after ART and significant decrease in CD14^+^CD16^+^ monocytes, approaching levels of PWoH by 12 months. Correlation of CD14^+^CD16^–^ monocytes with plasma viral load (*P* = 0.03, *r* = 0.39). (**D**) Negative correlation of CD14^–^CD16^+^ monocytes with plasma viral load (*P* = 0.0001, *r* = –0.65). Statistical significance was determined using 1-way ANOVA with Tukey’s test for multigroup comparisons. **P < 0.01 compared with HIV^–^ and pre-ART visits.

**Figure 3:**
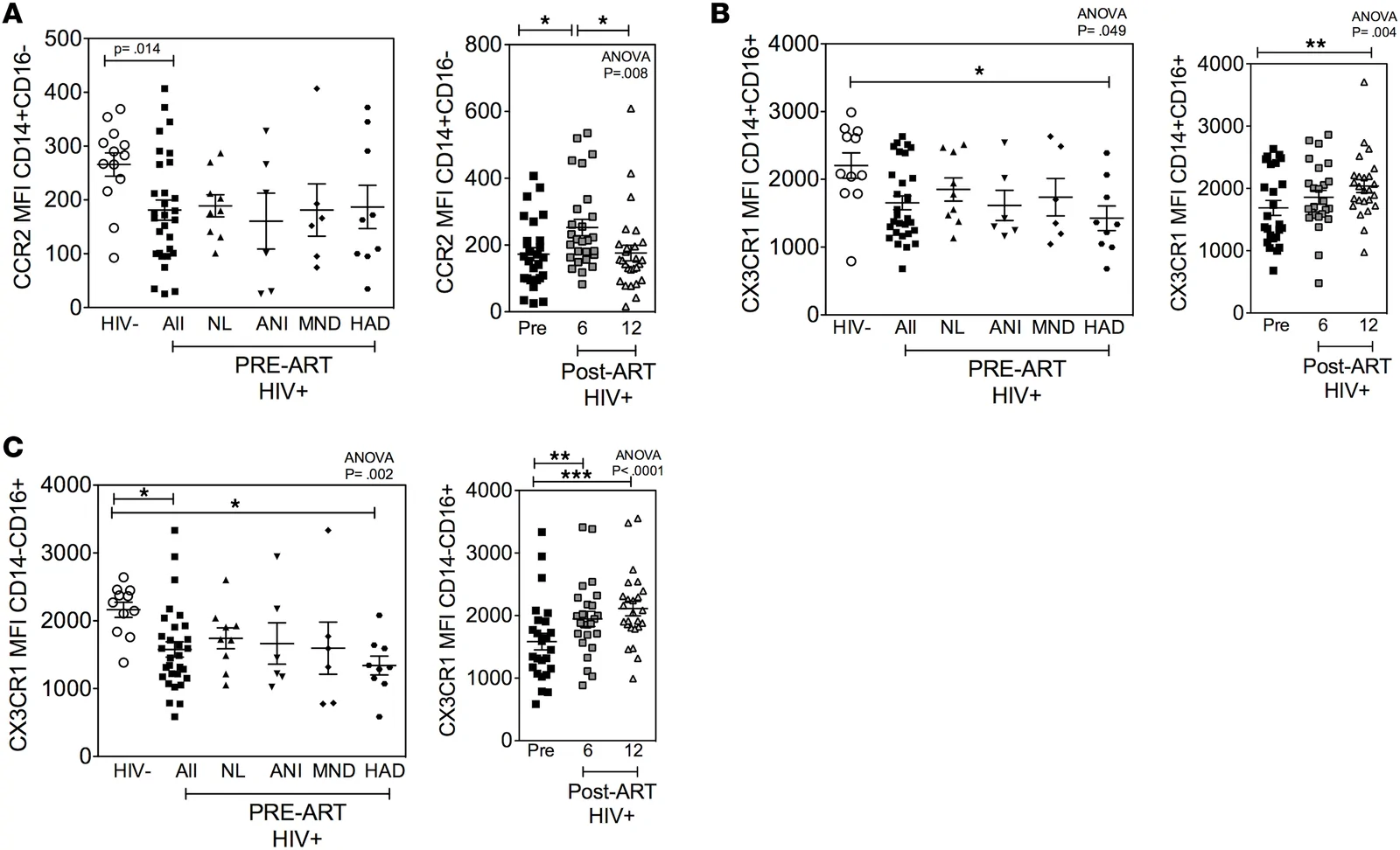
CCR2 and CX3CR1 expression on monocyte subsets before and after ART. (**A**) MFI of CCR2 on CD14^+^CD16^–^ classical monocytes was lower in PLWH before ART and increased significantly 6 months after ART before declining at 12 months. (**B** and **C**) CX3CR1 MFI on CD14^+^CD16^+^ (**B**) and CD14^–^CD16^+^ monocytes (**C**) with reduced expression in HIV^+^ individuals compared with HIV^–^. PLWH with HAD displayed the lowest CX3CR1 expression, which significantly increased after ART. (**C**) Significant increase in CX3CR1 MFI on CD14^lo^CD16^+^ monocytes at 6 and 12 months after ART. Statistical significance was determined using 1-way ANOVA with Tukey’s test for multigroup comparisons. *P < 0.05, **P < 0.01, ***P < 0.001 compared with PWoH and pre-ART visits.

**Figure 4:**
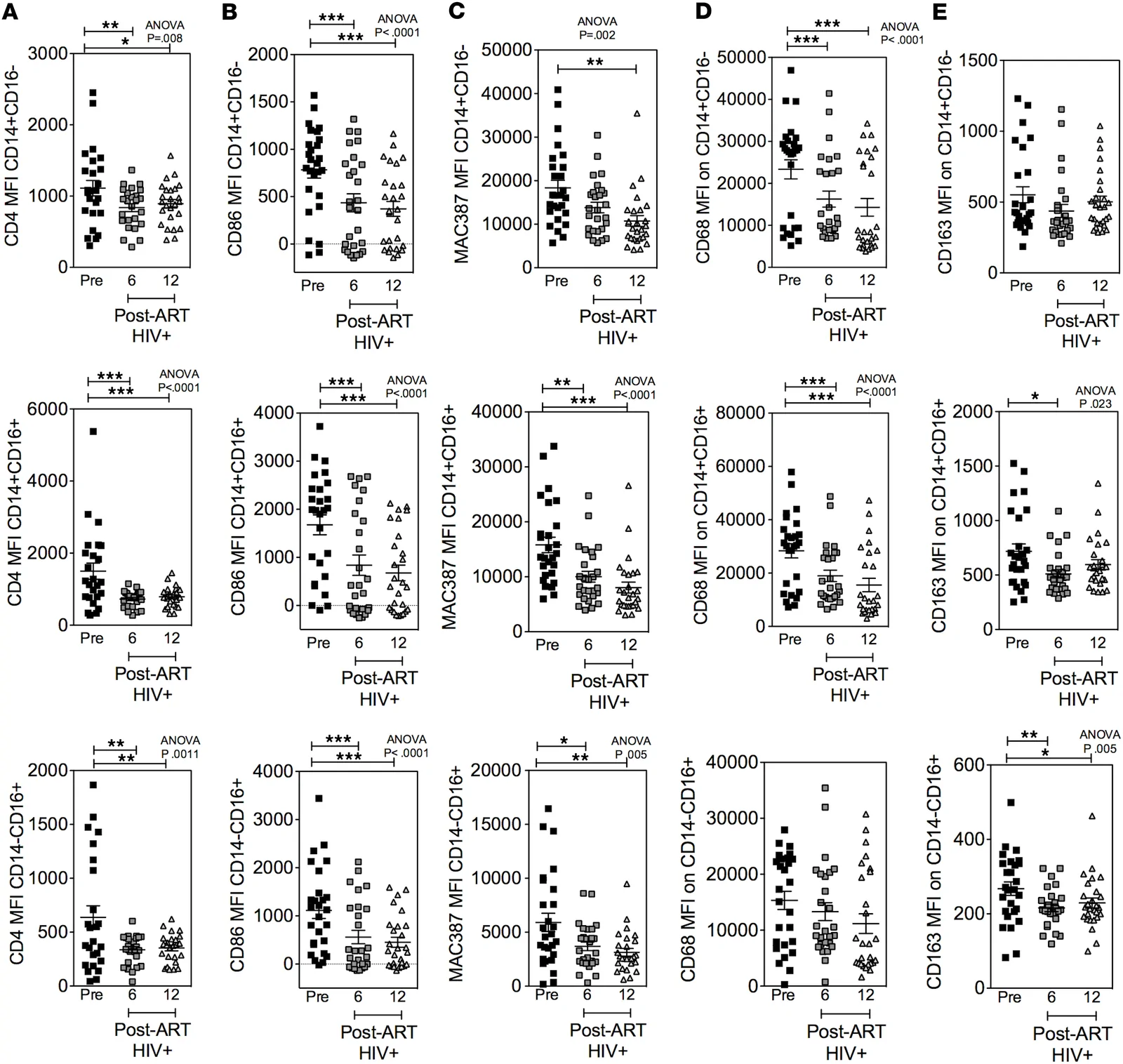
Cluster of differentiation marker expression on monocyte subsets before and after ART. (**A**–**E**) Decreased expression of CD4, CD86, MAC387, CD68 and CD163 on monocyte subsets following 6 and 12 months of ART. No significant changes were found in CD44v6 and CCR5 expression across any monocyte subsets before and after ART (data not shown). Statistical significance was determined using 1-way ANOVA with Tukey’s test for multigroup comparisons. *P < 0.05, **P < 0.01, ***P < 0.001 compared with pre-ART visits.

**Figure 5:**
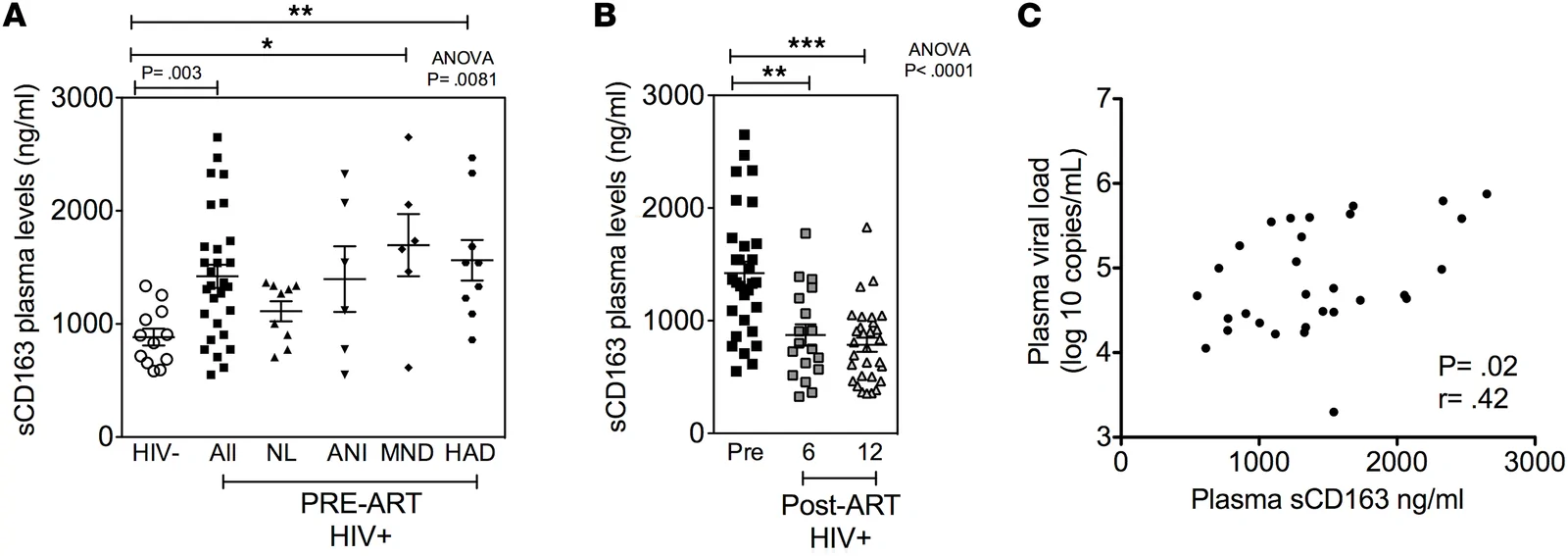
Plasma soluble CD163 levels before and after ART. (**A**) Elevated soluble CD163 (sCD163) levels in ART-naive PLWH compared with PWoH controls, especially in neurocognitively symptomatic individuals (MND and HAD) compared with asymptomatic (NL, ANI) individuals. (**B**) Significant reduction of sCD163 levels after 6 and 12 months of ART. (**C**) Correlation of sCD163 levels with plasma viral load before ART. Statistical significance was determined using 1-way ANOVA with Tukey’s test for multigroup comparisons. *P < 0.05, **P < 0.01, ***P < 0.001 compared with HIV^–^ and pre-ART visits.

**Figure 6:**
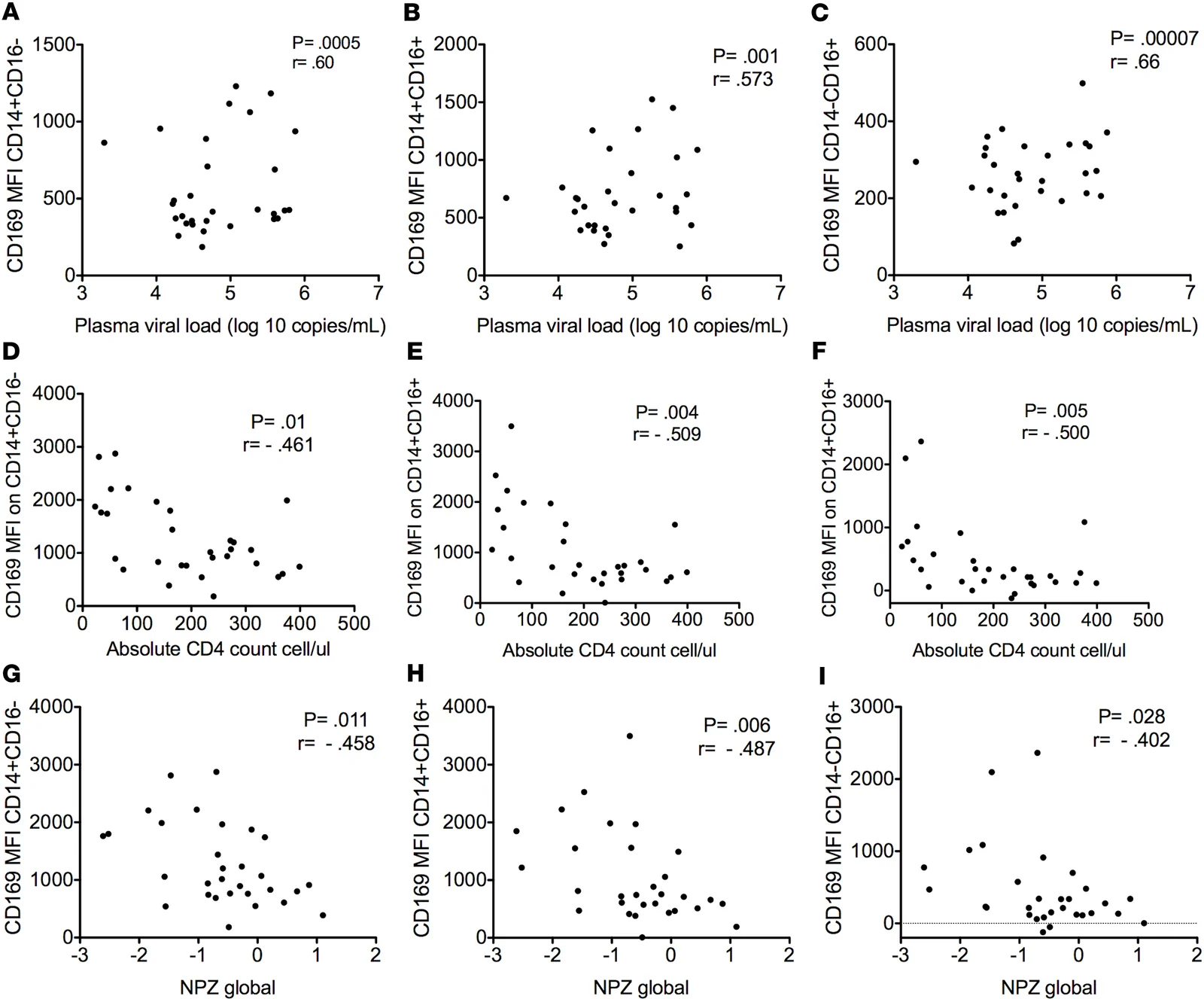
CD169 expression on monocyte subsets and correlations with plasma viral load and neurocognitive impairment. (**A**–**C**) MFI of CD169 on classical, intermediate, and inflammatory monocytes positively correlates with plasma viral load in ART-naive PLWH (*P* < 0.01 for all). (**D**–**F**) Negative correlation between monocyte CD169 MFI and CD4^+^ T cell counts. (**G**–**I**) Negative correlation between CD169 MFI on monocyte subsets and NPZglobal scores.

**Figure 7:**
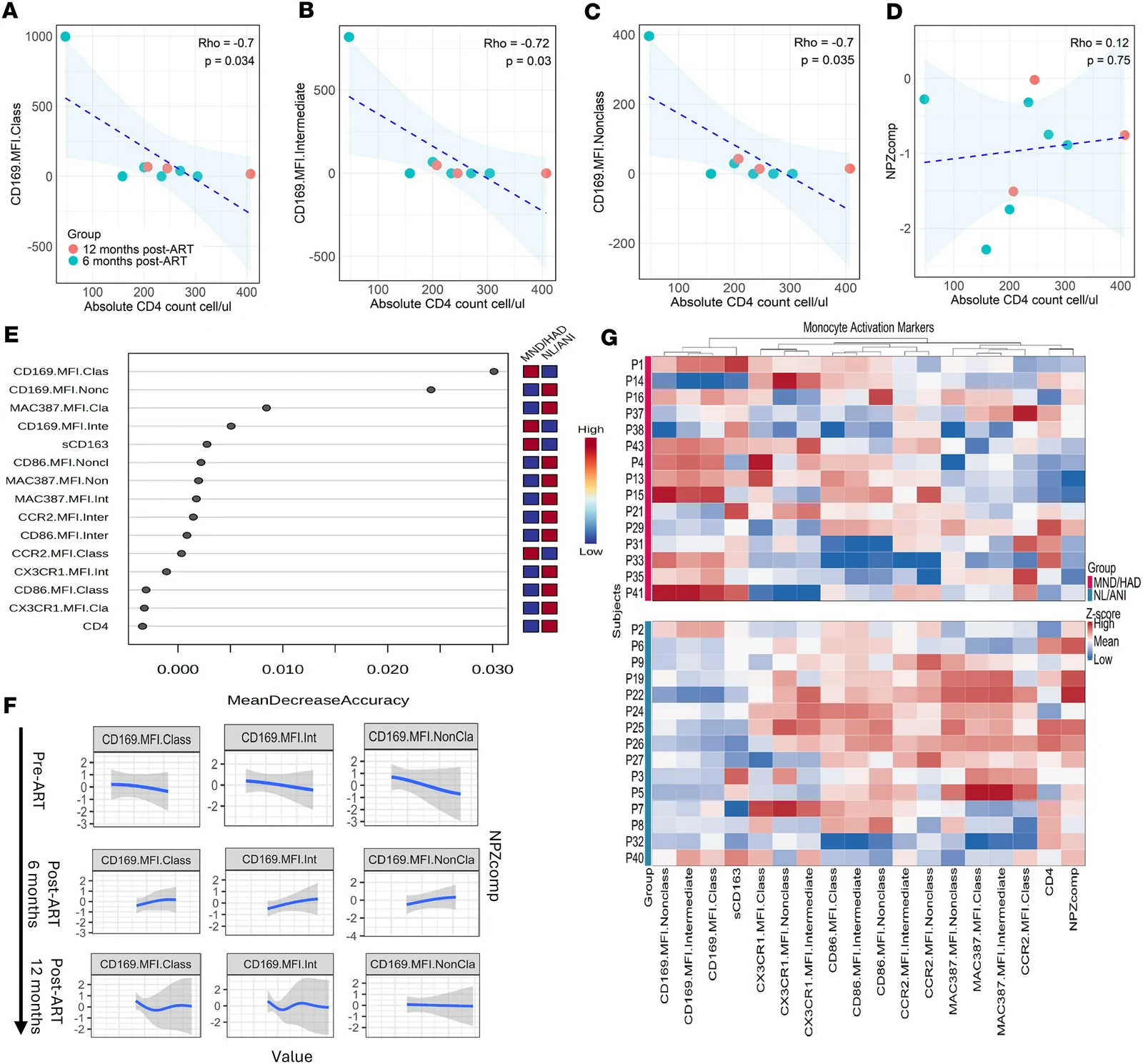
CD169 expression on monocyte subsets is associated with cognitive impairment compared with CD4^+^ T cell count, plasma viral load, and monocyte activation markers linked to HAND. (**A**–**D**) Spearman’s correlation analysis showing correlations between CD169 MFI on CD14^+^CD16^–^, CD14^+^CD16^+^, and CD14^lo^CD16^+^ monocytes and CD4^+^ T cell count. Analyses included 7 patients (P003, P004, P005, P013, P014, P015, and P016) who achieved viral suppression (plasma viral load < 50 copies/mL) but were diagnosed with ANI, MND, or HAD at 6 and 12 months after ART. (**E**) Random forest model identifying key predictors of cognitive impairment. The top 15 features contributing most to classification accuracy in distinguishing individuals with MND or HAD from those with normal cognition (NL) or ANI are shown. A higher mean decrease in accuracy indicates greater variable importance. The model used data from 30 patients with CD169 MFI measurements at baseline (pre-ART). (**F**) Bayesian kernel machine regression (BKMR) analysis evaluating nonlinear associations between CD169 expression on monocyte subsets, CD4^+^ T cell count, plasma viral load, monocyte activation markers (sCD163, CCR2, CD86, CX3CR1, and MAC387), and NPZcomp score at baseline and 6 and 12 months after ART. Models were adjusted for age and sex using data from 30 patients with CD169 MFI measurements. Increased CD169 expression was associated with lower NPZcomp scores when other variables were held at median levels. (**G**) Heatmap showing CD169 expression on monocyte subsets, CD4^+^ T cell count, plasma viral load, and monocyte activation markers associated with HAND using baseline data from 30 patients with CD169 MFI measurements.

**Figure 8:**
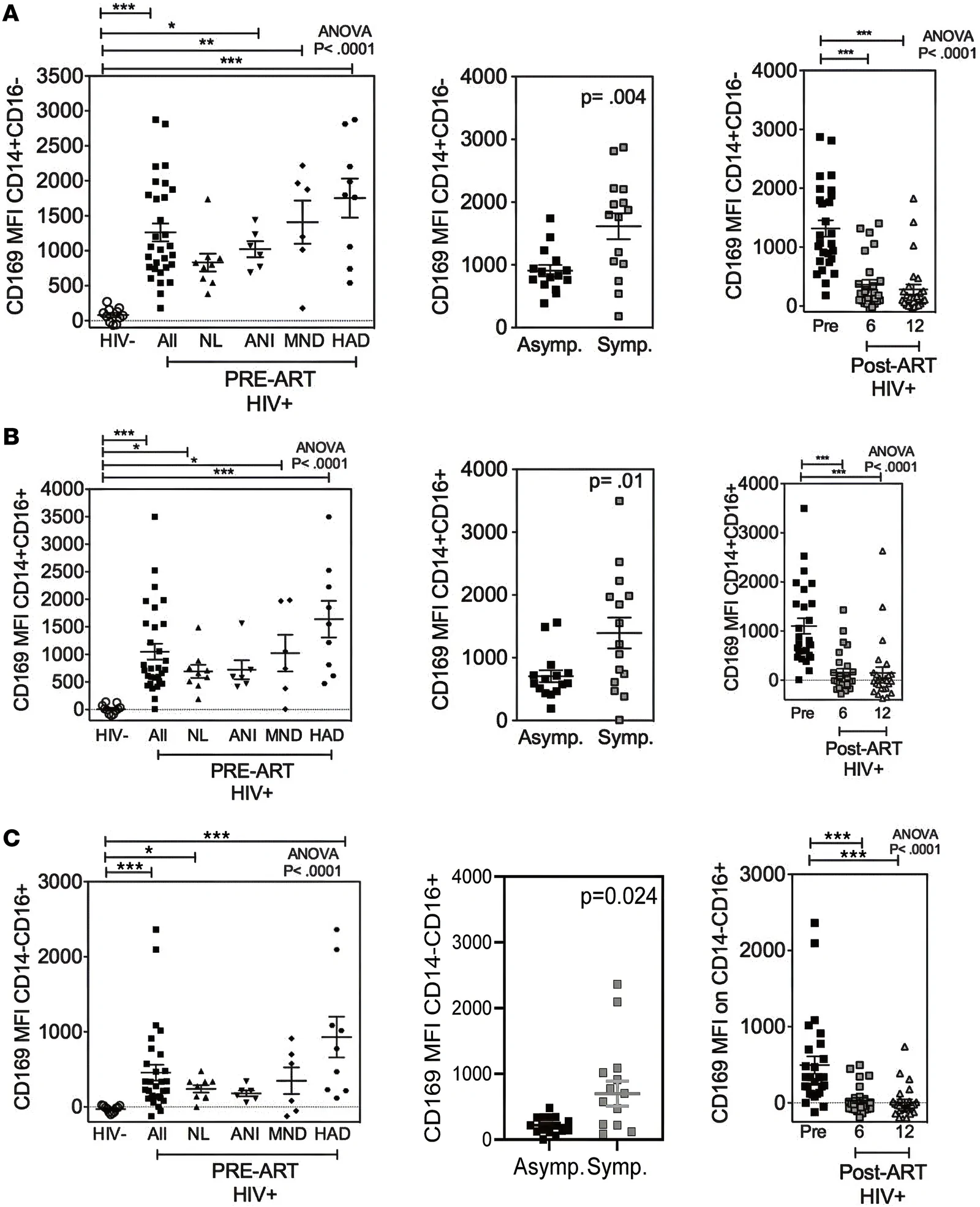
CD169 expression across HAND groups before ART. (**A**–**C**) Stepwise increase in MFI of CD169 on classical, intermediate, and inflammatory monocytes across HAND groups ranging from NL to HAD before ART. Significant differences were observed between asymptomatic (NL, ANI) and symptomatic (MND, HAD) patients in all monocyte subsets. Statistical significance was determined using Student’s *t* test and 1-way ANOVA with Tukey’s post hoc test for multigroup comparisons. *P < 0.05, **P < 0.01, ***P < 0.001 compared with HIV^–^ and pre-ART visits.

**Figure 9:**
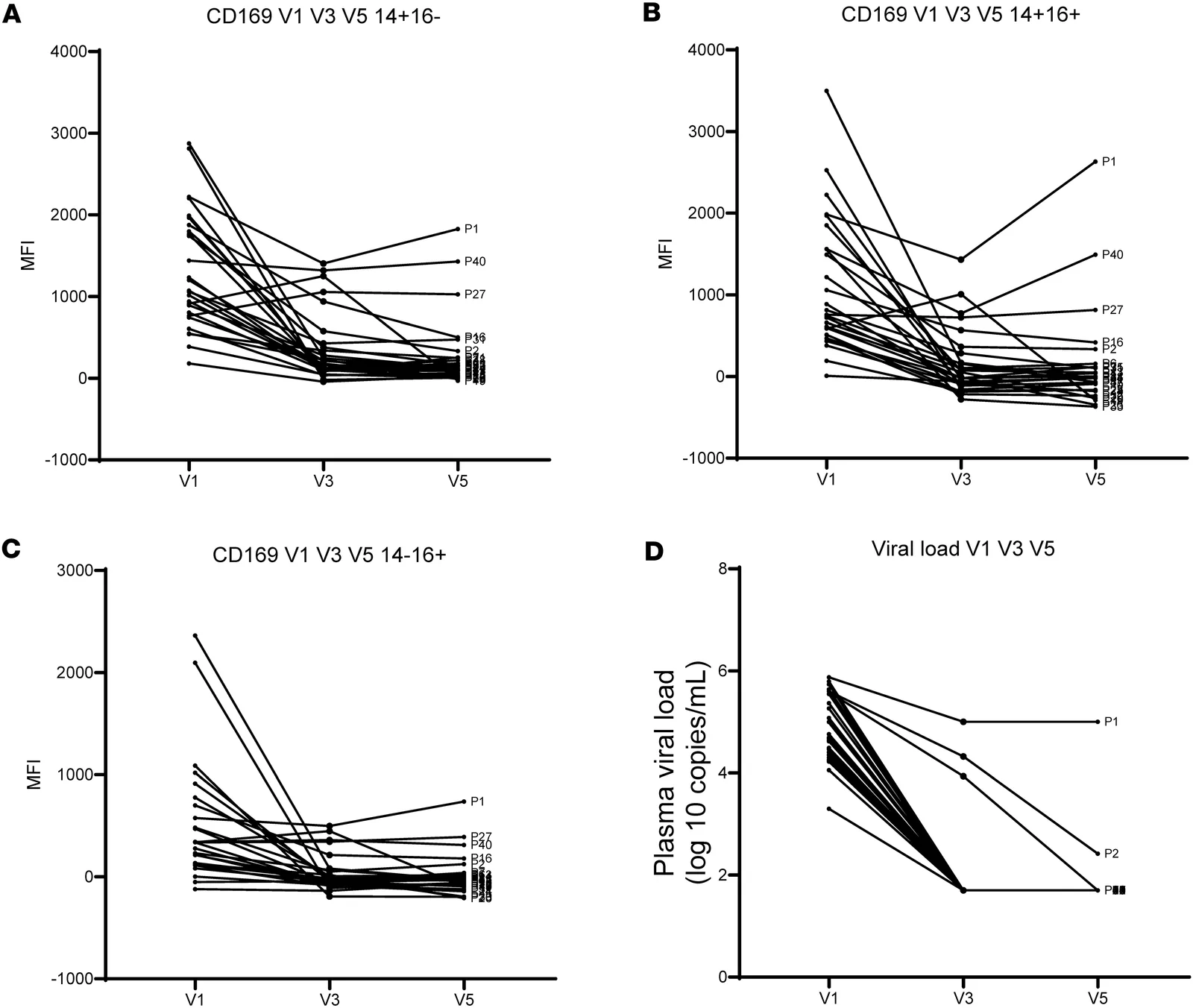
CD169 expression and viral load after ART. (**A**–**C**) Significant decreases in CD169 MFI on all monocyte subsets were observed at 6 and 12 months after ART on CD14^–^CD16^+^, CD14^+^CD16^+^, and CD14^–^CD16^+^ monocytes. V1, pre-ART; V2, 6 months post-ART; V5, 9 months post-ART. (**D**) Decreased plasma virus over time with ART. With ART, CD169 expression dropped to levels comparable to those of PWoH controls in most individuals examined. Non-virologically-suppressed individuals (P1, P2) had no significant changes in CD169 expression. The majority of virologically suppressed individuals exhibited a decrease in plasma viral load.

## Version 1 08/27/2026

In-Press Preview

## Version 2 10/08/2026

Electronic publication
