# Supplementary material for: Monocyte correlates of neurocognitive impairment in chronic HIV infection include early, persistent changes with ART
Source: JCI Insight. 2026 Aug 27;11(19):e194766. doi: 10.1172/jci.insight.194766 (PMC13726430; doi:10.1172/jci.insight.194766)
Supplement: Supplementary file 1 [file jciinsight-11-194766-s133.pdf]

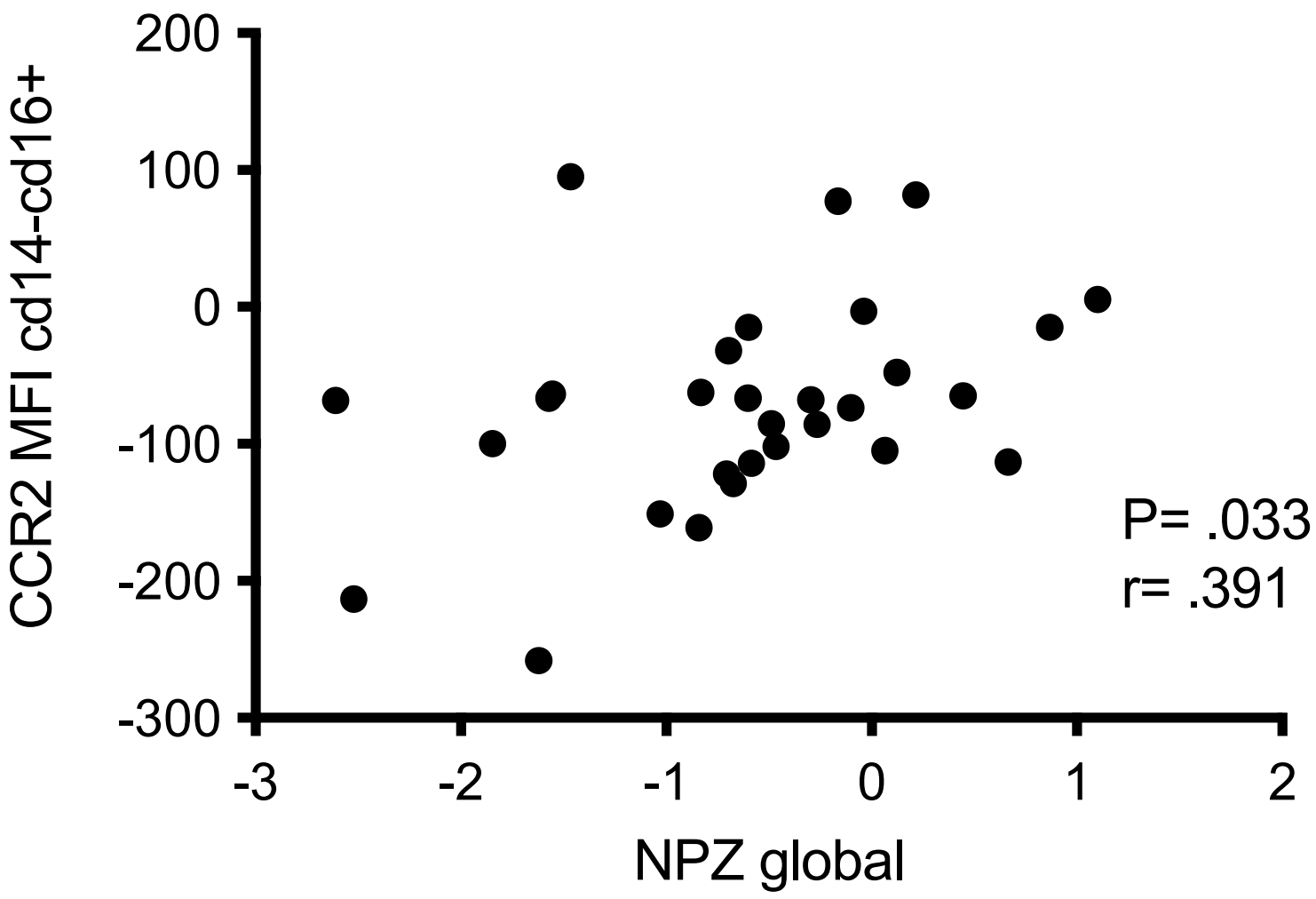

Figure S1. Correlation between CCR2 expression on classical monocytes and NPZ global score.

Spearman's correlation analysis showing a positive correlation (r=0.39, p=0.033) between the mean fluorescence intensity (MFI) of CCR2 on CD14+ monocytes and the NPZ global score in HIV+ individuals. CCR2 MFI was measured at baseline (pre-ART) and post-ART at 6 and 12 months. The NPZ global score represents neurocognitive function, with higher scores indicating better performance. The increase in CCR2 MFI on classical monocytes following ART was associated with improvements in neurocognitive performance.

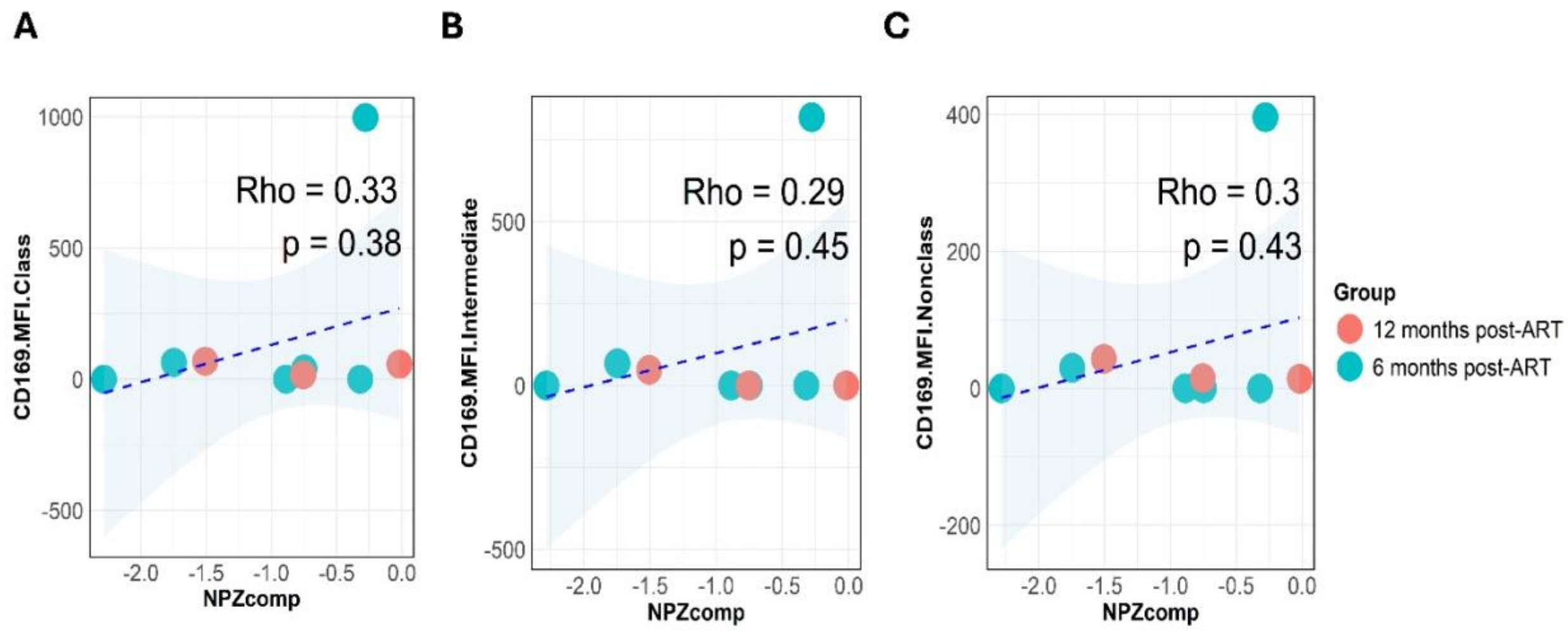

Figure S2. No significant correlation between CD169 expression on monocyte subsets and NPZcomp score in seven individuals with suppressed plasma viral load but diagnosed with HAND (ANI, MND, or HAD) at 6- and 12-months post-ART. in this small group of individuals with suppressed plasma viral load but with cognitive impairment, CD169 levels did not show a direct linear correlation with NPZcomp scores at any of the individual time points. Data were analyzed from seven patients (P003, P004, P005, P013, P014, P015, and P016) who achieved viral suppression (plasma viral load <50 copies/mL) but were diagnosed with asymptomatic neurocognitive impairment (ANI), mild neurocognitive disorder (MND), or HIV-associated dementia (HAD) at 6 and 12 months post-ART. Spearman's correlation analysis shows no significant correlation between the mean fluorescence intensity (MFI) of CD169 on three monocyte subsets and the NPZcomp score in HIV+ individuals who achieved viral suppression but were diagnosed with ANI, MND, or HAD. CD169 MFI was measured post-ART at 6 and 12 months. The NPZcomp score represents neurocognitive function, with higher scores indicating better performance. The increase in CD169 MFI on monocyte subsets following cART was not associated with improvements in neurocognitive performance in these specific individuals (p-value>0.05). CD169.Class: CD169 on CD14+CD16-; CD169. Intermediate: CD169 on CD14+CD16+; and CD169.Nonclass: CD169 on CD14lowCD16+.

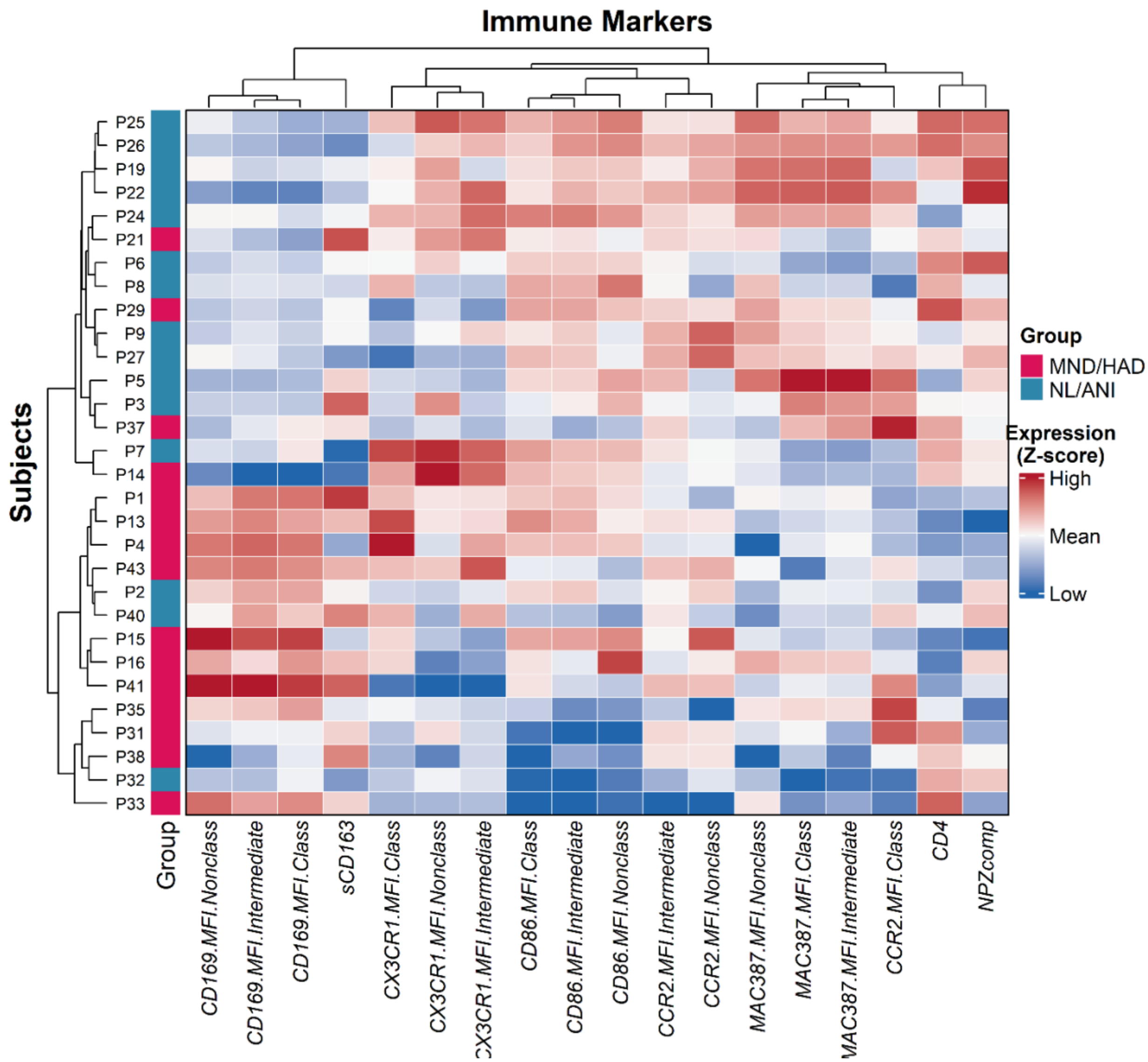

Figure S3. Heat map of CD169 expression on monocyte subsets, CD4+ T cell count plasma viral load, and markers of monocyte activation associated with HAND using data from 30 patients with measured CD169 MFI values. MND: mild neurocognitive disorder, HAD: HIV-associated dementia, NL: normal cognition, ANI: asymptomatic neurocognitive impairment (ANI).

A. **Model 1:** CD169.MFI.Class, PVL, and CD4 +T cell count, and monocyte activation markers associated with HAND (sCD163, CCR2, CD86, CX3CR1, and MAC387)

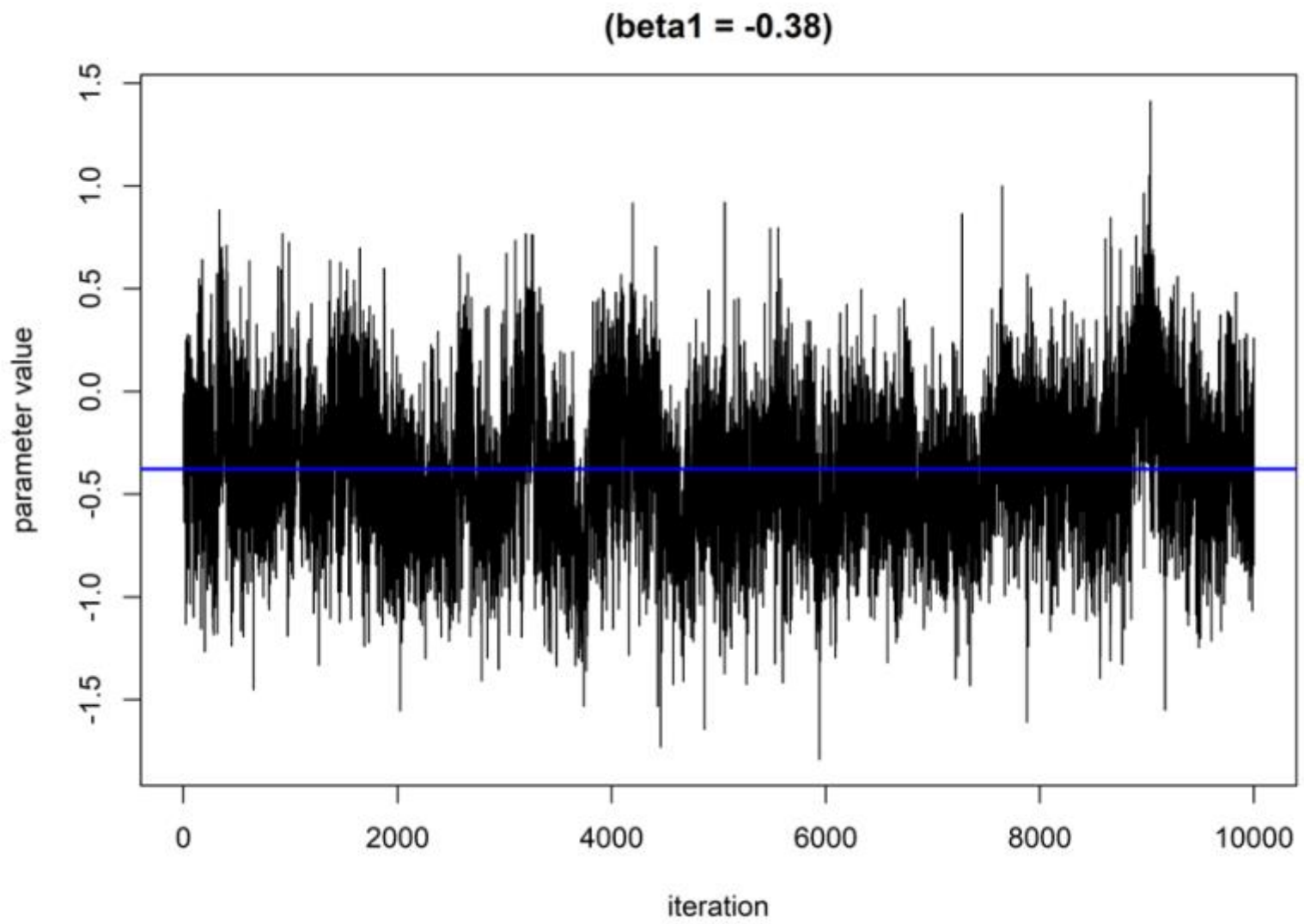

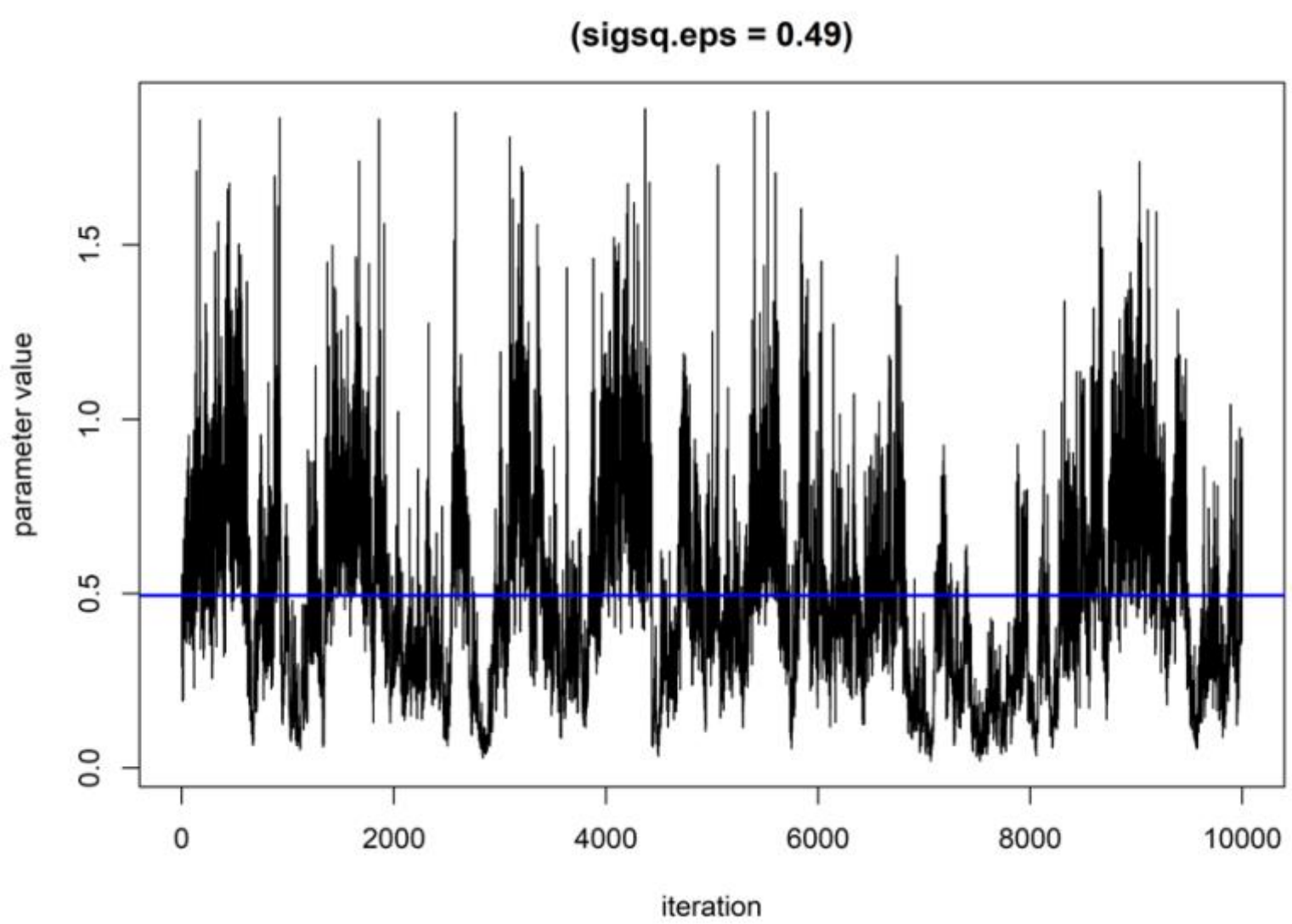

	Variable	PIP
1	**CD169.MFI.Class**	**0.721**
2	PVL	0.339
3	CD4	0.4634
4	sCD163	0.4088
5	CCR2.MFI.Class	0.4707
6	CCR2.MFI.Intermediate	0.4039
7	CCR2.MFI.Nonclass	0.4675
8	MAC387.MFI.Class	0.4806
9	MAC387.MFI.Intermediate	0.5151
10	MAC387.MFI.Nonclass	0.6399

11	CD86.MFI.Class	0.534
12	CD86.MFI.Intermediate	0.4892
13	CD86.MFI.Nonclass	0.4001
14	CX3CR1.MFI.Class	0.4579
15	CX3CR1.MFI.Intermediate	0.3937
16	CX3CR1.MFI.Nonclass	0.408

B. Model 2: CD169.MFI.Intermediate, PVL, and CD4 +T cell count, and other markers (CD86, CXCR2, CX3CR1, sCD163, and MAC387)

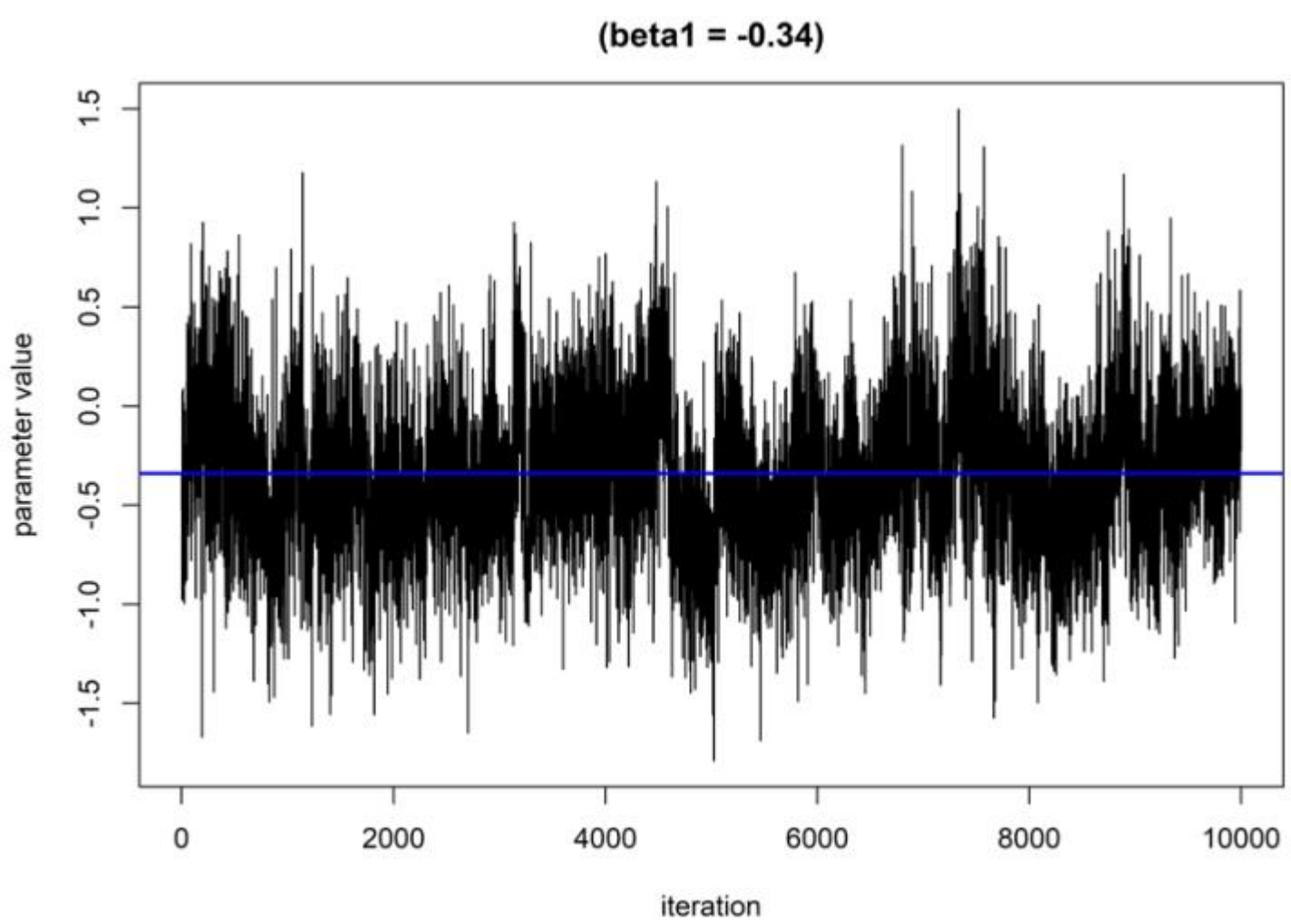

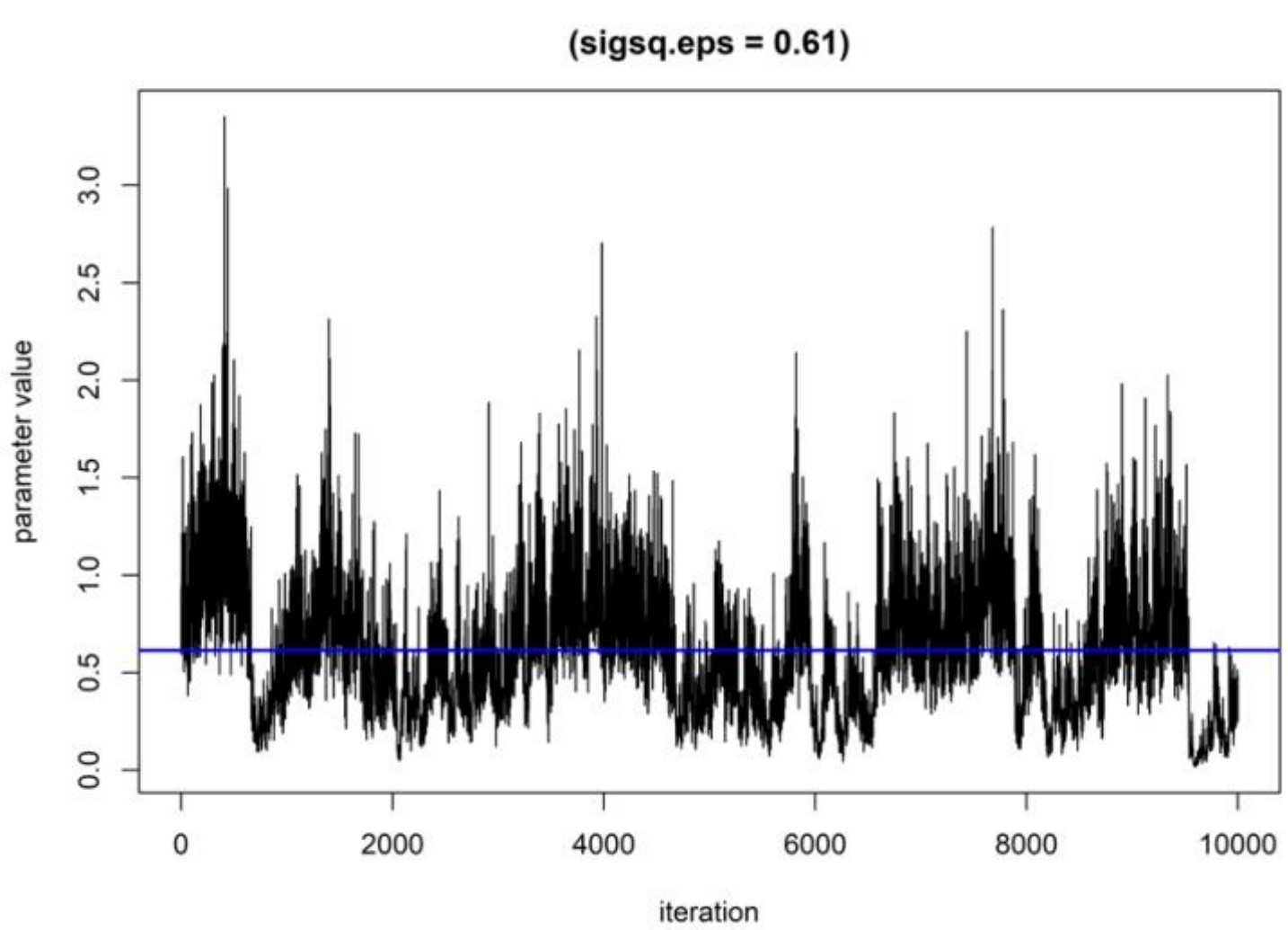

	variable	PIP
1	**CD169.MFI.Int**	**0.654**
2	PVL	0.4056
3	CD4	0.6419
4	sCD163	0.4291

5	CCR2.MFI.Class	0.5582
6	CCR2.MFI.Int	0.4766
7	CCR2.MFI.Noncla	0.5055
8	MAC387.MFI.Class	0.5594
9	MAC387.MFI.Int	0.5465
10	MAC387.MFI.Noncla	0.6745
11	CD86.MFI.Class	0.6443
12	CD86.MFI.Int	0.5615
13	CD86.MFI.Noncla	0.4436
14	CX3CR1.MFI.Class	0.5123
15	CX3CR1.MFI.Int	0.4
16	CX3CR1.MFI.Noncla	0.4165

C. **Model 3:** CD169.MFI.Nonclass, PVL, and CD4 +T cell count, and other markers (CD86, CXCR2, CX3CR1, sCD163, and MAC387)

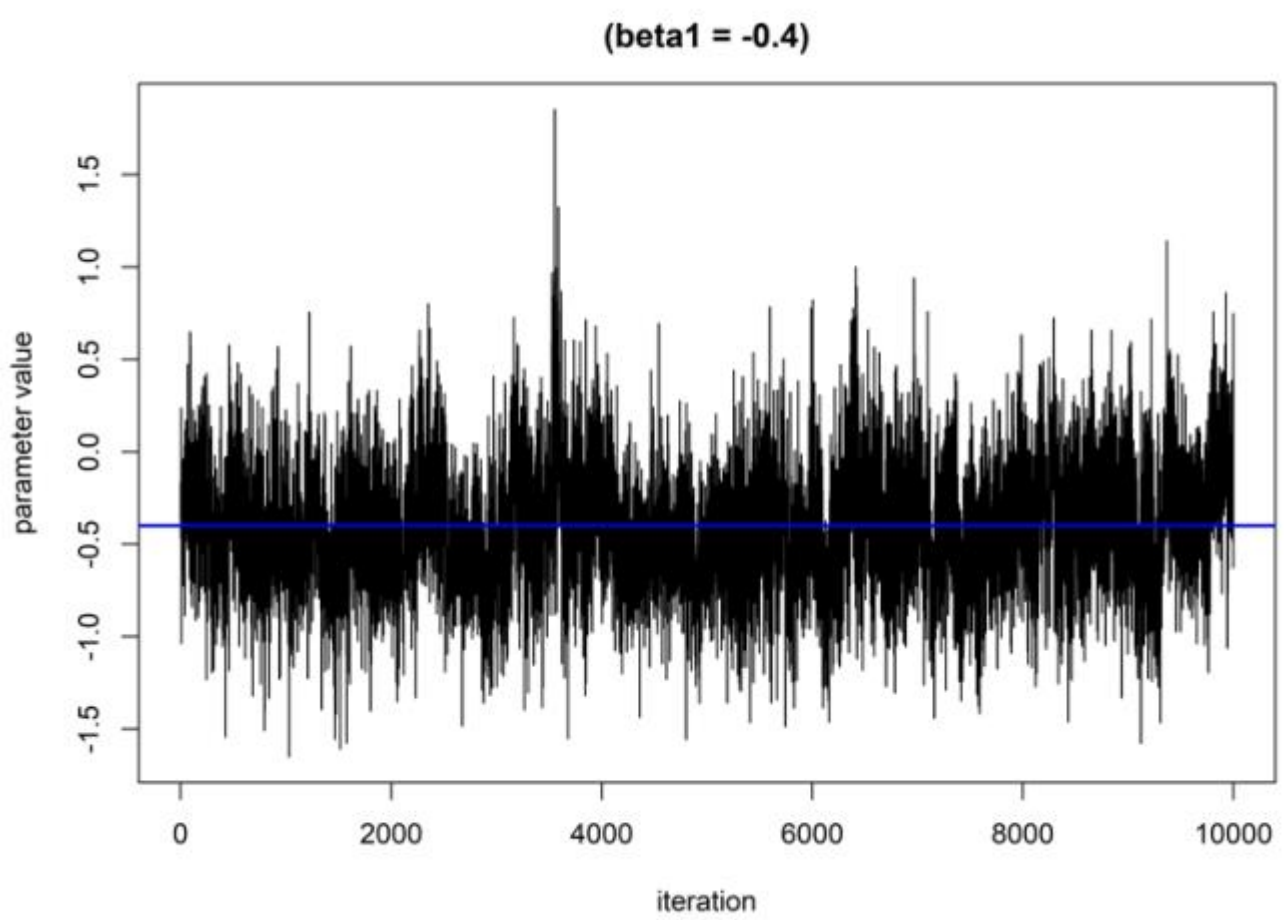

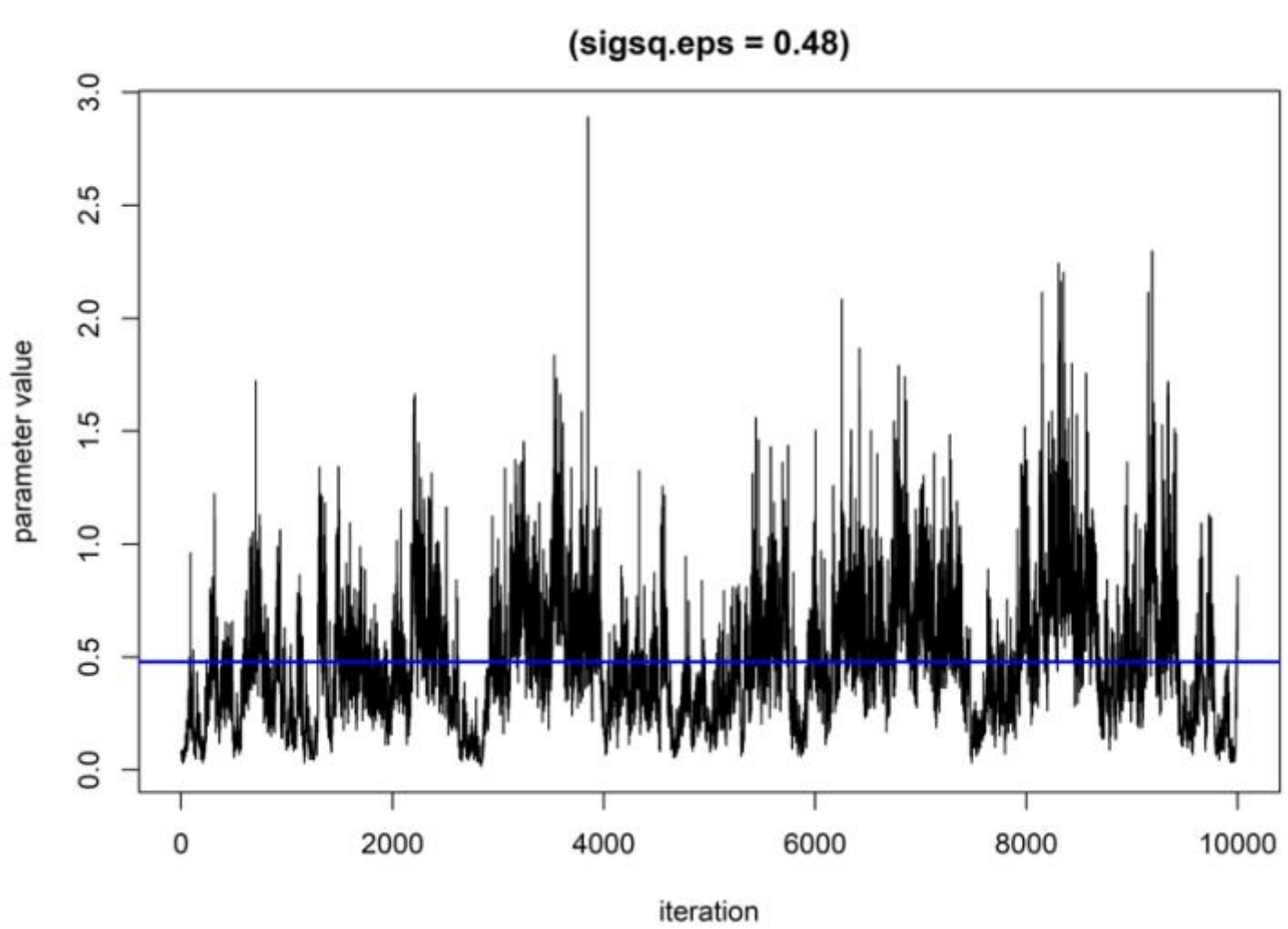

	variable	PIP
1	**CD169.MFI.NonCla**	**0.66**
2	PVL	0.4919
3	CD4	0.7375
4	sCD163	0.6197
5	CCR2.MFI.Class	0.7303
6	CCR2.MFI.Int	0.5499
7	CCR2.MFI.Noncla	0.7049
8	MAC387.MFI.Class	0.6481
9	MAC387.MFI.Int	0.6953
10	MAC387.MFI.Noncla	0.734
11	CD86.MFI.Class	0.8145
12	CD86.MFI.Int	0.7469
13	CD86.MFI.Noncla	0.5596
14	CX3CR1.MFI.Class	0.625
15	CX3CR1.MFI.Int	0.5664
16	CX3CR1.MFI.Noncla	0.5121

Figure S4. Beta (β), standard error (signse.eps), and posterior inclusion probability (PIP) for three BKMR models.

(A) Model 1: CD169.MFI.Class, plasma viral load (PVL), CD4+ T cell count, and other markers (CD86, CXCR2, CX3CR1, sCD163, and MAC387).

(B) Model 2: CD169.MFI.Intermediate, PVL, CD4+ T cell count, and other markers (CD86, CXCR2, CX3CR1, sCD163, and MAC387).

(C) Model 3: CD169.MFI.Nonclass, PVL, and CD4+ T cell count, and other markers (CD86, CXCR2, CX3CR1, sCD163, and MAC387).

BKMR models were adjusted for age and sex using data from 30 patients. Beta (β) represents the influence of each predictor in the model. Signse.eps likely refers to the significance or standard error of the model's residuals, indicating how well the model fits the data and the uncertainty associated with the predicted relationships. A posterior inclusion probability (PIP) threshold of 0.5 was used to determine the importance of each factor.

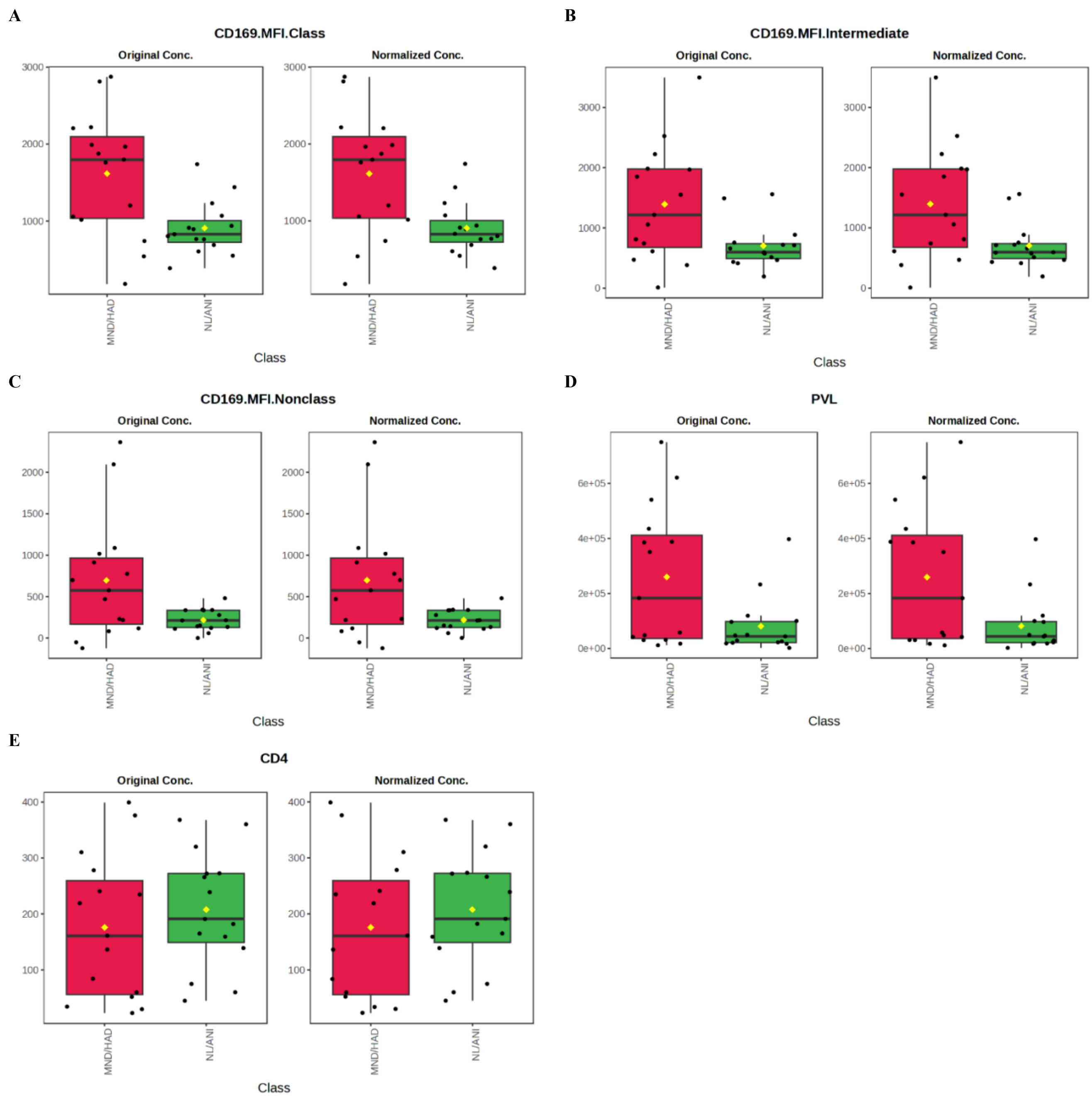

Figure S5. Random forest model identifies key predictors of cognitive impairment. A random forest model was applied to identify the most important variables associated with cognitive impairment in people with HIV, using data from 30 patients with measured CD169 MFI values. Boxplots display the distributions of selected predictors, shown as both raw (filtered count) and $\log_2$-transformed values. Each boxplot indicates the median (center line), interquartile range (IQR; box), and full data range (whiskers). CD169 expression on monocyte subsets and plasma viral load were elevated in individuals with mild neurocognitive disorder (MND) or HIV-associated dementia (HAD), compared to those with normal cognition (NL) or asymptomatic neurocognitive impairment (ANI). In contrast, $CD4^+$ T cell counts were higher in the NL/ANI group, suggesting

an inverse relationship with cognitive impairment severity. ART status was defined based on visit time points: pre-ART (no ART), 6 months post-ART, and 12 months post-ART (on ART).

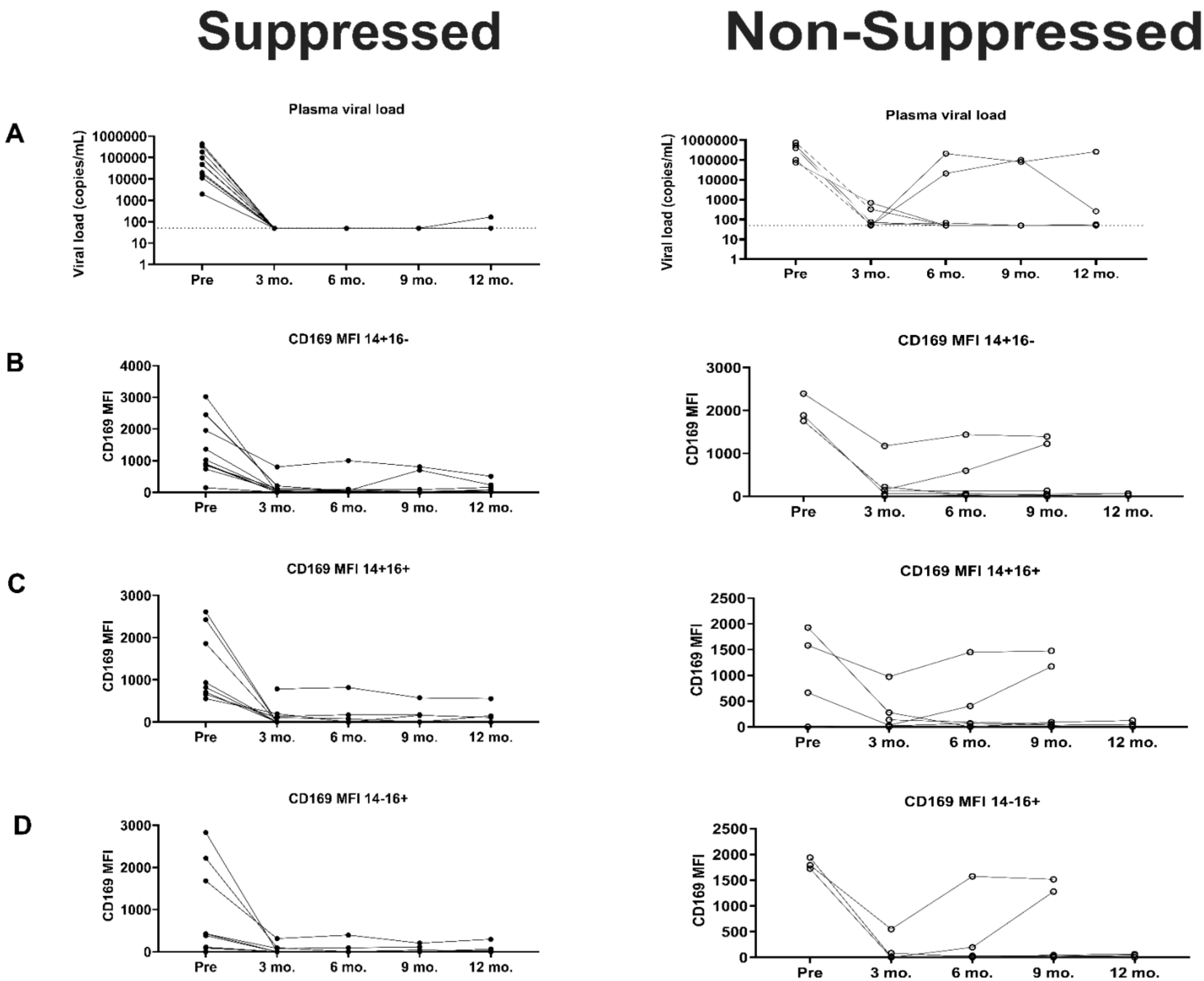

Figure S6. Changes in viral load and monocyte subset frequencies.
(A) Plasma viral load in the suppressed and non-suppressed groups across timepoints (pre-ART and 3, 6, 9, and 12 months post-ART initiation).
(B) Percentage of $CD14^{+}CD16^{-}$ monocytes in the suppressed and non-suppressed groups across timepoints (pre-ART and 3, 6, 9, and 12 months post-ART initiation).
(C) Percentage of $CD14^{+}CD16^{+}$ monocytes in the suppressed and non-suppressed groups across timepoints (pre-ART and 3, 6, 9, and 12 months post-ART initiation).

(D) Percentage of CD14^lowCD16^{+} monocytes in the suppressed and non-suppressed groups across timepoints (pre-ART and 3, 6, 9, and 12 months post-ART initiation).
